# Biophysical characterization of the recording of unmyelinated and myelinated fiber activity with peripheral interfaces

**DOI:** 10.1016/j.isci.2025.112495

**Published:** 2025-04-22

**Authors:** Claudio Verardo, Simone Romeni, Silvestro Micera

**Affiliations:** 1 The Biorobotics Institute and Department of Excellence in Robotics and AI, Scuola Superiore Sant’Anna, Pisa, Italy; 2 Modular Implantable Neuroprostheses (MINE) Laboratory, Università Vita-Salute San Raffaele & Scuola Superiore Sant’Anna, Milan, Italy; 3 Bertarelli Foundation Chair in Translational Neural Engineering, Neuro-X Institute, Ecole Polytechnique Federale de Lausanne, Lausanne, Switzerland

**Keywords:** Neuroscience, Cell biology, Biophysics

## Abstract

Unmyelinated fibers account for a remarkable fraction of the peripheral nervous system and their activity is linked to many autonomic and somatic functions. While electrical recording of such activity from human-sized peripheral nerves holds significant potential for neuroengineering applications, it has been shown only in acute settings via microneurography. This leaves unclear whether current implantable electrodes could achieve the same outcome. To address this matter, we simulated recordings from the human vagus nerve through a transverse intrafascicular multichannel electrode (TIME), a microneurographic (μNG) needle, and a commercial cuff electrode. Recording signals were studied fiber-wise across relevant electrode insertions, revealing that the possibility of recording unmyelinated activity is shared by the TIME but unlikely by the cuff. These results suggest that no physical limitations of implantable electrodes underlie the missing evidence of recordings from unmyelinated fibers, and draw attention to experimental design choices that may have concealed this capability thus far.

## Highlights

• We characterized *in silico* the recording strength of relevant peripheral electrodes
• Intraneural recording strongly depends on the fiber-electrode relative position
• Intrafascicular insertion enables large amplitude recording of unmyelinated fibers
• Extraneural recording may not be well-suited for transducing unmyelinated activity

## Graphical abstract

**Figure:**
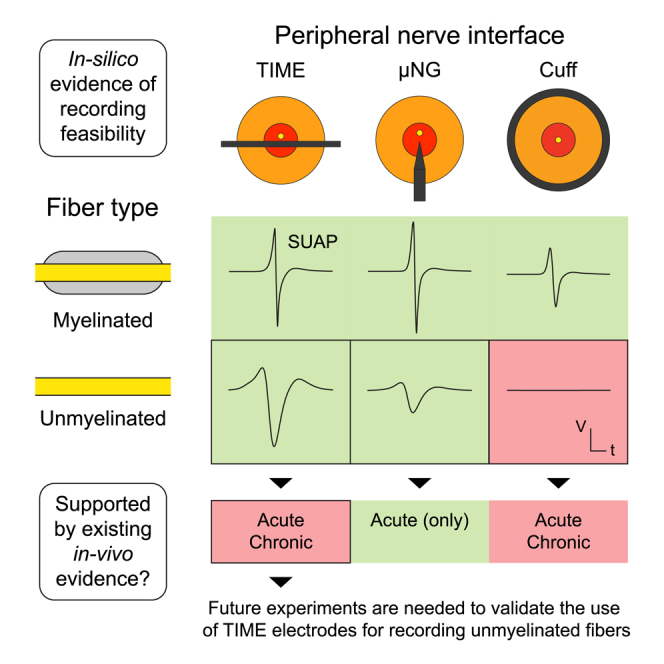

## Introduction

Recording of physiologically evoked neural activity from electrodes implanted in peripheral nerves has recently received attention as it may serve to provide feedback signals for the closed-loop neuromodulation of bodily functions,^1^ to control the actuation of artificial limb prostheses or functional electrical stimulation (FES) systems,^2^ and to develop novel biomimetic stimulation protocols through the understanding of neural encoding.^3^

The recording of autonomic nerves is of primary interest given their involvement in the control of many bodily functions. For example, vagus nerve recordings have been successfully linked to immune,^4^^,^^5^ metabolic,^6^^,^^7^ cardiovascular,^8^^,^^9^ and respiratory^10^^,^^11^ functions in animal models, with the modulations of cardiovascular and respiratory functions confirmed in human subjects as well.^12^^,^^13^ Instead, recordings from the pudendal nerve have been used to infer volume and pressure of the bladder in preclinical studies.^14^^,^^15^ Among somatic nerves, recordings of the neural activity through the median and ulnar nerves have been employed to decode attempted motor tasks in amputees^16^^,^^17^^,^^18^ and sensory information in spinal cord injury subjects.^19^

In all of these nerves, information flows through several distinct fiber populations, ranging from large caliber myelinated fibers to very thin unmyelinated fibers, with the latter largely surpassing in number the former in many cases^20^ (see Table S1). Capturing the activity of unmyelinated fibers through implanted electrodes has a significant translational potential, as their discharge patterns have been linked to the immune, metabolic, cardiovascular, and respiratory systems in autonomic nerves,^21^^,^^22^ and to noxious, thermal, and affective tactile sensations in somatic nerves.^23^ Nonetheless, despite positive evidence in recordings from small-sized animal models with both extraneural^24^ and intraneural^6^ electrodes, no work has proven the translatability to human-sized peripheral nerves. This is even more striking considering the diversity of the recording electrodes employed, including cuffs,^10^^,^^11^ transverse intrafascicular multichannel electrodes (TIMEs),^8^^,^^14^^,^^16^ longitudinal intrafascicular electrodes (LIFEs),^18^^,^^25^ and Utah slanted electrode arrays (USEAs).^17^ Instead, microneurographic (μNG) needles acutely inserted in human somatic and autonomic nerves have shown successful recording of unmyelinated fibers encoding, for instance, affective tactile sensation,^26^ and cardiac activity.^13^

Achieving effective recording of unmyelinated activity in translational settings requires a clear understanding of the fiber-wise signal strength transduced by peripheral implants in human-sized peripheral nerves, particularly in comparison to μNG needles. This is not feasible with the current body of experimental evidence, as it is not possible to rule out many uncontrolled confounding factors at play during recording sessions that could have hindered the optimal observation of such activity. For example, autonomic nerve studies in large-sized animals^25^^,^^27^ typically involve anesthetics that are known to alter neural activity,^28^^,^^29^ and the few works using less disruptive anesthetics have not attempted to identify the unmyelinated origin of the detected fibers (e.g., through conduction velocity analysis).^9^^,^^10^ On the other hand, although recordings from somatic nerves have been performed in awake humans,^16^^,^^17^^,^^18^ no experimental protocols tailored to excite unmyelinated fibers have been performed, contrary to microneurography studies.^26^ Electrode insertion represents a further confounder in the case of intraneural electrodes designed for clinical translation, like TIMEs.^2^ Indeed, while these electrodes can in principle be placed close to the target fibers, their blind surgical implantation potentially reduces the likelihood of achieving intrafascicular insertion in the multifascicular nerves of large-sized animals.^27^^,^^30^ As a result, their transduction potential might be underestimated compared to microneurography studies, wherein the insertion is guided by real-time monitoring of the recorded neural activity.

Computational models of peripheral nerve implants enable to investigate neural recordings at the level of single fibers switching off all possible experimental confounders, and have thus the potential to shed light on how unmyelinated activity translates into recording signals across different electrodes in the same experimental conditions. The current modeling approach is referred to as hybrid modeling,^31^ and has been introduced for peripheral nerve recordings in the study by Andreasen et al.^32^ It exploits multicompartmental axon models to simulate the electrogenic activity of nerve fibers and finite-element modeling (FEM) to translate nerve fiber transmembrane currents into simulated recorded signals. Most computational works focus on extraneural electrodes,^32^^,^^33^^,^^34^^,^^35^^,^^36^^,^^37^^,^^38^^,^^39^^,^^40^^,^^41^^,^^42^ with only a few taking into account the activity of unmyelinated fibers^34^^,^^41^^,^^42^ but restricting to murine-sized nerve geometries. On the contrary, all of the works modeling intraneural electrodes, such as TIME,^40^^,^^43^ LIFE,^44^ and μNG,^45^ consider only the activity of myelinated fibers, following the widely adopted assumption that the recording component associated with unmyelinated fibers is negligible.^40^ Therefore, previous *in-silico* studies are not sufficient to assess the possibility of recording unmyelinated activity with current implantable electrodes in human-sized peripheral nerves.

In this work, we hypothesize that the ability to record unmyelinated fibers from human-sized peripheral nerves is not a unique feature of μNG electrodes but can also be achieved with implantable electrodes, provided that the same experimental conditions are used. To test this, we employ computational modeling to characterize the amplitude of the waveforms that can be recorded from myelinated and unmyelinated fibers across different electrode types and insertion configurations. Specifically, we analyze the cases of a TIME and a μNG needle with intrafascicular or extrafascicular insertions for the class of intraneural electrodes, and a commercial cuff electrode for the class of extraneural ones. The same nerve structure is employed in all the analyses to isolate the effects of fiber type, electrode geometry, and electrode insertion on the recording signal.

## Results

### Modeling workflow and case studies of peripheral recording

**Figure 1:**
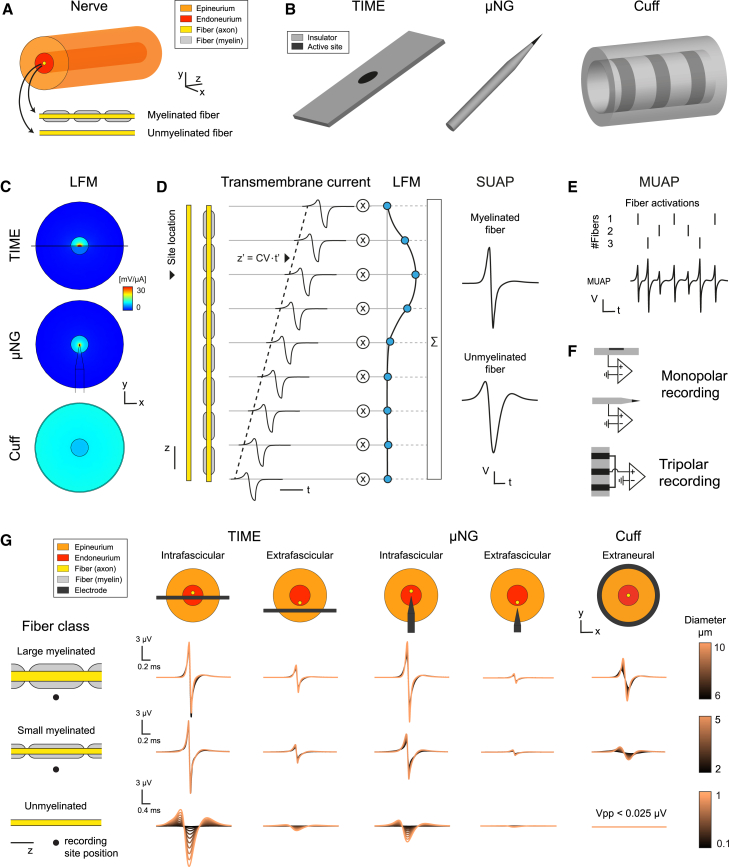
Modeling workflow and case studies of peripheral nerve recording (A) Nerve geometry. (B) Electrodes geometries for TIME, μNG, and cuff. (C) Lead field matrix (LFM) calculation using finite-element modeling (FEM). (D) Single-unit action potential (SUAP) of a fiber obtained by combining the transmembrane currents of the fiber nodes during the propagation of an action potential with the LFM of the recording site. (E) Multi-unit activity (MUAP) recordings simulated by convolving the activations of the fibers with their SUAPs. (F) Monopolar recordings are considered for the TIME and μNG, while tripolar recording for the cuff. (G) Combinations of electrode geometries and insertions (columns), and fiber classes (rows) considered as case studies in the work. For each combination, example SUAPs are shown with colors encoding the fiber diameters and electrode-to-fiber relative positions detailed in the text. The axes scale bars of each row are shown in the first column.

The modeling workflow adopted to simulate peripheral recordings is illustrated in Figure 1. Throughout our analyses, we employed a nerve model sized to the human cervical vagus nerve^46^ with a diameter of 2 mm containing a single fascicle with a diameter of 400 μm (Figure 1A), which has been placed in the center of the nerve section, if not specified otherwise. We compared intraneural recordings performed with a TIME or a μNG needle with extraneural recording through a commercial cuff (Figure 1B). The framework for these computational experiments is described in detail in the STAR Methods. Briefly, after building the lead field matrix (LFM) collecting the *trans*-resistances between the fiber nodes and a given active site (Figure 1C), we linearly combined the transmembrane currents generated by the nodes of single fibers during the propagation of an action potential to produce single unit action potential (SUAP) waveforms (Figure 1D). The MRG model^47^ was used to describe the electrogenic activity of myelinated fibers, while the Sundt model^48^ was used for unmyelinated fibers. SUAPs from different fibers that are concurrently active can then be combined to produce a multi-unit signal (MUAP) (Figure 1E). While monopolar SUAPs have been considered for the TIME and the μNG, to adopt the same setup employed in microneurographic studies, tripolar SUAPs have been considered for the cuff, as this configuration is routinely used in experimental settings^10^^,^^25^ to reject non-neural signals^49^ (Figure 1F).

We employed our computational framework to characterize the signal recorded by various combinations of fiber types and electrode insertions (Figure 1G). We considered large-myelinated fibers (> 6 μm diameter), small-myelinated fibers (2–5 μm diameter), and unmyelinated fibers (0.1–1.5 μm diameter) representing Aβ, Aδ/B, and C fiber types from the Erlanger-Gasser classification^50^ as well as the main fiber populations in the vagus nerve^51^ (Figure 1G, rows). For intraneural electrodes (TIME and μNG), we compared the cases where the active site was inside the fascicle (intrafascicular insertion) or outside the fascicle (extrafascicular insertion), while for the cuff electrode, we considered an extraneural placement in direct contact with the nerve epineurium (Figure 1G, columns). Example SUAPs are shown for all these case studies (Figure 1G, insets), assuming a fiber-to-electrode distance of 20 μm for intraneural electrodes and with the fiber at the center of the fascicle for the cuff. In addition, for myelinated fibers, the recording site is located at half of the internode. Throughout the work, we positioned fibers 10 μm deep inside the fascicle when considering extrafascicular insertions, it is not otherwise specified. This value, representing the smallest perineurium thickness in the human vagus nerve,^46^ is adopted as a best-case scenario to investigate if recordings with sufficiently large amplitude are possible with extrafascicular insertions. We point out that this choice is not as critical in the cuff case since the *trans*-resistance between fiber and recording site is almost uniform inside the fascicle with the cuff, while it is highly influenced by the recording site position with intraneural electrodes (Figure 1C). Therefore, fibers will be always placed at the center of the fascicle when considering the cuff, if not otherwise specified.

### SUAP amplitudes exhibit complex dependence upon electrode-fiber relative position

**Figure 2:**
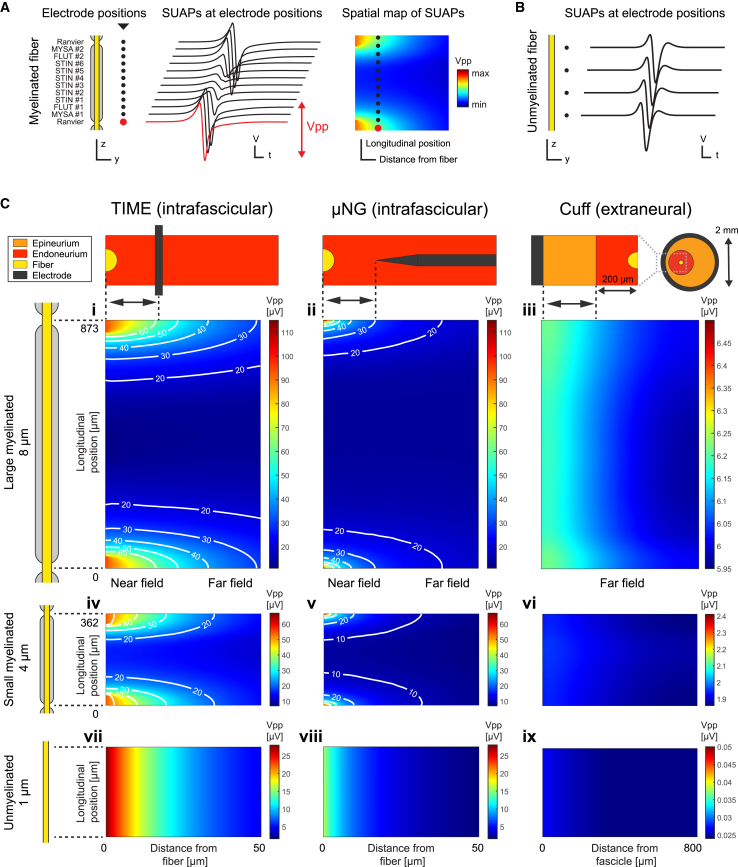
*Dependence of SUAP amplitudes upon the electrode-fiber relative position* (A) Illustration of the process to compute the spatial map of SUAP amplitudes for a given combination of electrode geometry, electrode insertion, and fiber type. The case of a myelinated fiber is considered. (B) The case of unmyelinated fiber is considered, showing that SUAPs do not depend upon the longitudinal position of the electrode. (C) Spatial maps of SUAP amplitudes. Rows refer to the recorded fiber types: 8 μm-myelinated, 4 μm-myelinated, and 1 μm-unmyelinated. Columns refer to the geometry and insertion of the recording electrode: intrafascicular TIME, intrafascicular μNG, and cuff.

We first investigated how the recording amplitude varies when we displace the electrode away from the fibers or along their path, by examining such spatial dependency in the SUAPs (Figure 2). In the case of myelinated fibers, moving the recording contact along the fiber produces a modification in the SUAP amplitude as the electrode passes close to nodes of Ranvier or internodes provided by the non-uniform expression of ion channels.^47^ In addition, increasing the distance between the recording contact and the fiber reduces the *trans*-resistance coupling between the two, thereby lowering the SUAP amplitude.^52^ Such two-dimensional dependence can be effectively visualized by plotting the SUAP peak-to-peak amplitude as a two-dimensional spatial map (Figure 2A). We represented the case unmyelinated fibers similarly, even though the SUAP amplitude does not vary when the recording electrode is displaced along the fiber (Figure 2B), given the uniform expression of ion channels.^48^ Further details regarding the generation of the SUAP spatial maps may be found in the STAR Methods.

Figure 2C shows the spatial maps of the SUAPs obtained for the intrafascicular insertion of the TIME and μNG, and the extraneural placement of the cuff, restricting the analysis to a large 8 μm myelinated fiber, a small 4 μm myelinated fiber, and a 1 μm unmyelinated fiber. While for intraneural electrodes the distance from the fiber is changed by moving the electrodes, for the cuff we chose to vary it by displacing the nerve fascicle. In the case of intrafascicular recording of myelinated fibers (Figure 2C, insets i–ii, iv–v), a strong longitudinal modulation of the amplitude of SUAPs can be seen for small electrode-to-fiber distances: we denote this phenomenon by calling it the “near-field region”. The maximum peak-to-peak is found in correspondence to fiber Ranvier nodes, while minimum is at the middle of the internode. Increasing the electrode-to-fiber distance, we enter a “far-field region” where such longitudinal modulation is not present anymore. Conversely, for the myelinated SUAPs recorded from the cuff (Figure 2C, insets iii and vi), we observe a mild dependence in the amplitude from both the longitudinal position of the electrode and its distance from the fiber. For unmyelinated fibers (Figure 2C, insets vii–ix), the SUAP amplitude only depends on the fiber-to-electrode distance, with intraneural electrodes exhibiting a steeper spatial decay compared to the cuff. Figure S1 shows the SUAP spatial maps in the case of extrafascicular insertion of the TIME and μNG, where near-field and far-field regions are present similar to the intrafascicular case, even though the amplitude modulation of the near-field regime is less pronounced and the peak-to-peak of SUAPs are considerably smaller.

### Near-field intraneural SUAPs of unmyelinated and myelinated fibers have similar amplitudes

**Figure 3:**
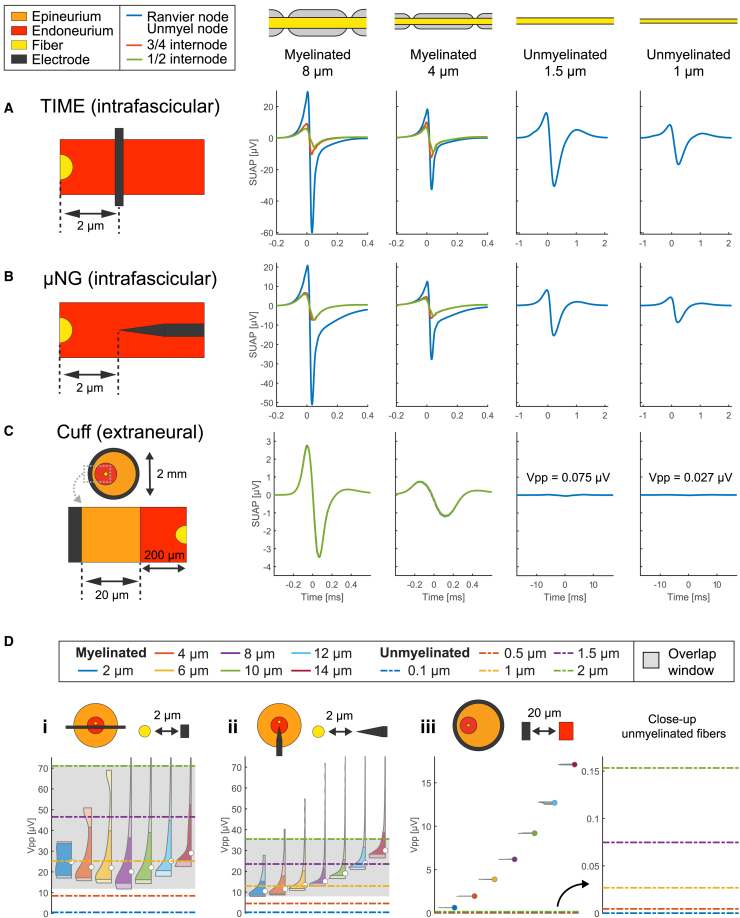
Relative amplitude of myelinated and unmyelinated SUAPs with electrodes close to fibers (A–C) Representative SUAPs are compared for (A) intrafascicular TIME, (B) intrafascicular μNG, and (C) cuff, at fiber-to-electrode distances detailed in the illustrations on the left of each panel. Columns correspond to fiber types: 8 μm-myelinated, 4 μm-myelinated, 1.5 μm-unmyelinated, and 1 μm-unmyelinated fibers. For myelinated fibers, the SUAPs are plotted at the Ranvier node, three-quarters of the internode, and half of the internode. (D) Generalization of the analysis to more fiber types, comparing the distributions of peak-to-peak amplitudes of SUAPs along each fiber. The shaded area corresponds to the range of amplitudes shared by myelinated and unmyelinated fibers. Data are represented as violin plots for myelinated fibers and lines for unmyelinated fibers.

We then expanded our analysis to study the relative amplitudes of the recordings from different fiber types. Given the complex spatial dependence of recording amplitudes, we conducted this study with electrodes placed both in close proximity to and at a greater distance from the target fibers, starting with the former case (Figure 3). We thus compared the SUAPs obtained in the relevant case studies of 8 μm-myelinated, 4 μm-myelinated, 1.5 μm-unmyelinated, and 1 μm-unmyelinated fibers (Figures 3A–3C). To account for the longitudinal dependence observed in the near-field region of myelinated fibers, we considered the SUAPs recorded at the Ranvier node, three-quarters of the internode, and half of the internode, which approximately yield the maximum, median, and minimum peak-to-peak amplitudes for the selected fiber-to-electrode distance (Figure S2). We note that for intrafascicular insertions (2 μm from the fiber, Figures 3A and 3B), unmyelinated fibers provide SUAPs with amplitudes that are comparable or even greater than those observed along myelinated fibers. Conversely, the cuff (20 μm from the fascicle, Figure 3C) exhibits myelinated SUAPs with a peak-to-peak amplitude 20 to 200-fold larger than their unmyelinated counterpart.

To gain further insight on these trends, we expanded the study to a larger repertoire of fiber types: from 2 μm to 14 μm-myelinated fibers and from 0.1 μm to 2 μm-unmyelinated fibers (Figure 3D). Specifically, we compared the distributions of peak-to-peak amplitudes of the SUAPs along the fibers, represented as violin plots for myelinated fibers and single data points for unmyelinated fibers. In the case of intrafascicular insertions (Figure 3D, insets i and ii), it exists an interval of peak-to-peak amplitudes shared by the SUAPs of both unmyelinated and myelinated fibers, which for this reason will be referred to as “overlap window” (shaded area). Such interval involves unmyelinated fibers with diameter greater than 0.5 μm and extends for several tens of μV. In the case of the cuff (Figure 3D, inset iii), we observe a distinct amplitude separation of the SUAPs across the degree of myelinization and the fiber diameters. When considering extrafascicular insertions (2 μm from the fascicle, Figure S3), an overlap window still exists, but it is limited to large unmyelinated fibers and extends for only a few μV.

### Far-field intraneural SUAPs of myelinated fibers have larger amplitudes than unmyelinated ones

**Figure 4:**
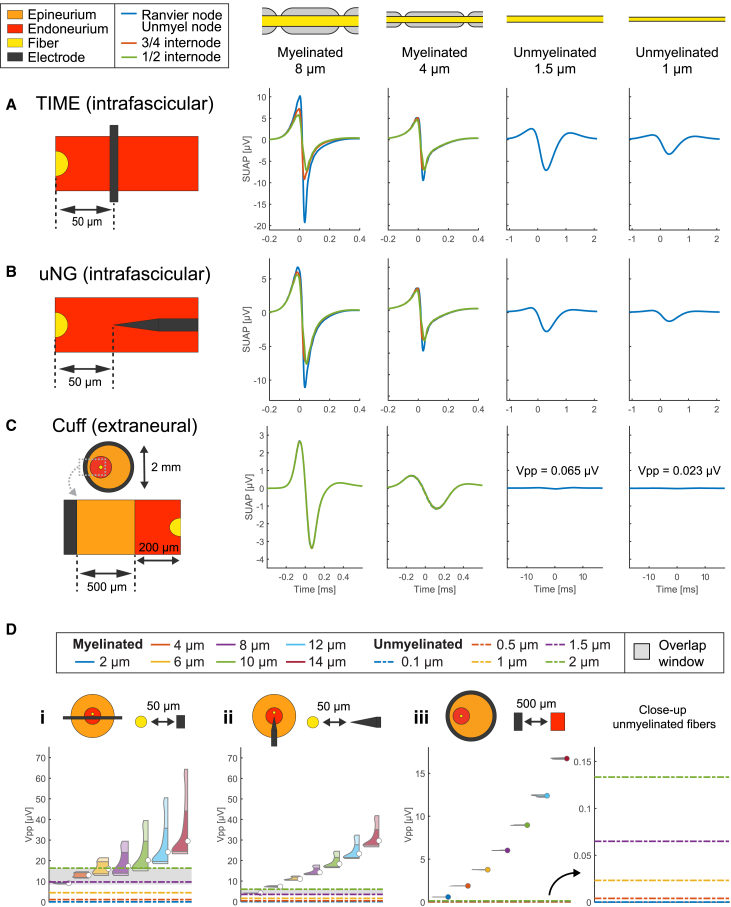
Relative amplitude of myelinated and unmyelinated SUAPs with electrodes far from fibers (A–C) Representative SUAPs are compared for (A) intrafascicular TIME, (B) intrafascicular μNG, and (C) cuff, at fiber-to-electrode distances detailed in the illustrations on the left of each panel. Columns correspond to fiber types: 8 μm-myelinated, 4 μm-myelinated, 1.5 μm-unmyelinated, and 1 μm-unmyelinated fibers. For myelinated fibers, the SUAPs are plotted at the Ranvier node, three-quarters of the internode, and half of the internode. (D) Generalization of the analysis to more fiber types, comparing the distributions of peak-to-peak amplitudes of SUAPs along each fiber. The shaded area corresponds to the range of amplitudes shared by myelinated and unmyelinated fibers. Data are represented as violin plots for myelinated fibers and lines for unmyelinated fibers.

We then investigated how moving the electrodes farther from the fibers affect the fiber-wise profile of relative recording amplitudes. Accordingly, we repeated the analyses from Figure 3 using larger electrode-to-fiber distances (Figure 4). For intrafascicular insertions (50 μm from the fiber, Figures 4A and 4B), we observe a weaker longitudinal dependence of the SUAPs of intraneural electrodes, due to the entering of the far-field regime. Unmyelinated and myelinated fibers have still comparable amplitudes, but the 1 μm and 1.5 μm-unmyelinated fibers have now SUAPs with peak-to-peak values smaller than those of 4 μm and 8 μm-myelinated fibers (compare with Figures 3A and 3B). Conversely, the waveforms collected by the cuff (500 μm from the fascicle, Figure 4C) closely resemble those obtained for small fiber-to-electrode distance, with only slightly smaller amplitudes (compare with Figure 3C).

Expanding the study to include more fiber types (Figure 4D) reveals a more pronounced segregation of unmyelinated fibers to lower peak-to-peak amplitudes in the intrafascicular case (Figure 4D, insets i and ii). Consequently, the overlap window is now much narrower and limited to large (> 1.5 μm) unmyelinated fibers and small (< 6 μm) myelinated fibers (compare to Figure 3D, insets i and ii). In addition, a clearer amplitude separation of SUAPs starts to emerge across myelinated fibers. In contrast, the results for the cuff (Figure 4D, inset iii) confirm almost unaltered the profile of amplitude separation that was observed for smaller fiber-to-electrode distance (compare to Figure 3D, inset iii). Similar to the intrafascicular case, extrafascicular insertions (50 μm from the fascicle, Figure S4) witness to the near disappearance of the overlap window, along with a more distinct fiber-wise amplitude segregation.

### Intrafascicular implantation is required to effectively transduce unmyelinated fibers

**Figure 5:**
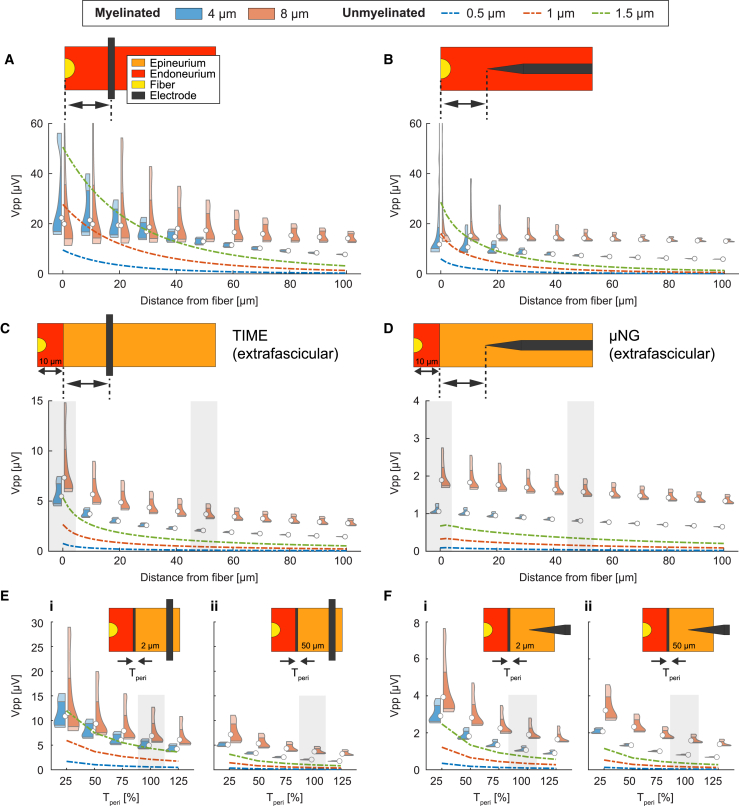
Factors influencing the fiber-wise recording amplitude in intraneural electrodes The longitudinal distributions of peak-to-peak amplitudes of SUAPs are considered while varying the fiber-to-electrode distance with (A) intrafascicular TIME, (B) intrafascicular μNG, (C) extrafascicular TIME, and (D) extrafascicular μNG. Further, the effect of varying the perineurium thickness is explored with (E) extrafascicular TIME and (F) extrafascicular μNG, for two relevant fiber-to-electrode distances. Shaded areas in (E) and (F) identify simulations performed under the same conditions in (C) and (D), respectively. In all panels, results are compared across two myelinated fibers (4 and 8 μm) and three unmyelinated fibers (0.5, 1, and 1.5 μm). Data are represented as violin plots for myelinated fibers and lines for unmyelinated fibers.

To gain deeper insight into the trends observed for recording amplitudes with intraneural electrodes, we compared the distribution of SUAPs peak-to-peak values between two myelinated fibers (diameters of 4 μm and 8 μm) and three unmyelinated fibers (diameters of 0.5 μm, 1 μm, and 1.5 μm) at a fine discretization of fiber-to-electrode distances (Figure 5). For intrafascicular insertions (Figures 5A and 5B), both myelinated and unmyelinated fibers exhibit SUAP amplitudes larger than 10 μV across tens of micrometers of electrode-fiber distance. With extrafascicular insertions (Figures 5C and 5D), such signal strength is obtained only for large-myelinated fibers with the TIME placed in close vicinity to the fascicle boundary. This trend persists in the case of thinner perineurium sheaths (Figures 5E and 5F), where we note that halving the value used as baseline, based on the human vagus nerve,^46^ results in a perineurium thickness approximately equal to 3% of the fascicle diameter, which is typical of somatic nerves.^53^ We also note that, for intrafascicular insertions (Figures 5A and 5B), the onset of the fiber-wise amplitude separation occurs at smaller distances with the μNG compared to the TIME (about 30 μm vs. 60 μm, respectively). This trend is emphasized for extrafascicular insertions (Figures 5C and 5D), where an amplitude separation is always present for the μNG, while it appears at about 10 μm distance with the TIME.

### Cuff electrodes exhibit large recording amplitude only for compound action potentials

**Figure 6:**
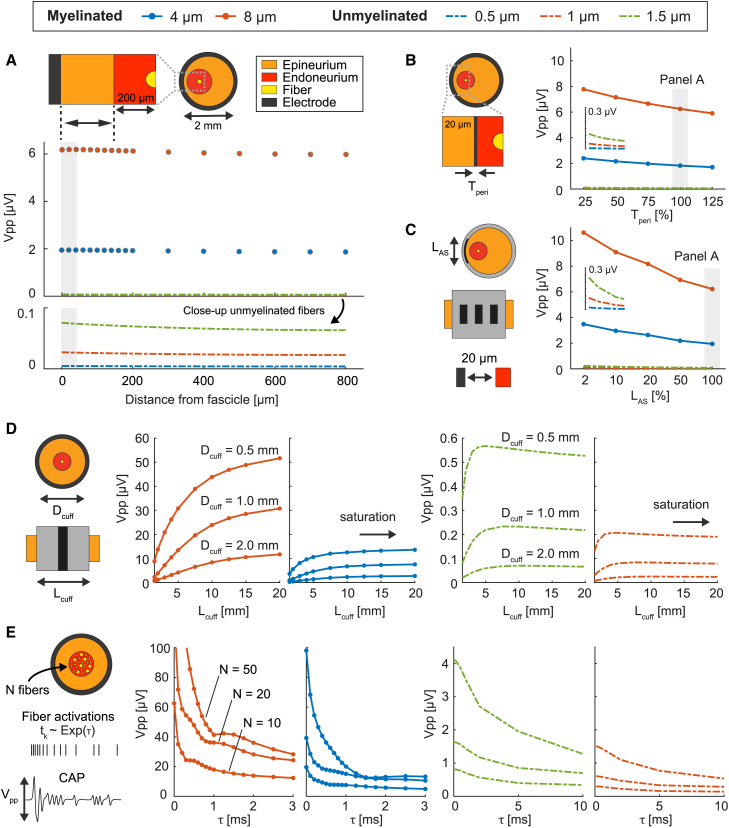
Factors influencing the fiber-wise recording amplitude in cuff electrodes (A–C) (A) The peak-to-peak amplitudes of SUAPs are considered while varying the fiber-to-electrode distance. For a fixed fiber-to-electrode distance, the effects of (B) perineurium thickness and (C) coverage of the active sites are explored. Shaded areas in (B) and (C) identify simulations performed under the same conditions in (A). (D) Amplitude of the SUAPs obtained for different cuff (and nerve) diameters and cuff lengths. (E) Amplitude of the compound action potentials (CAPs) obtained for different numbers of active fibers (N) and synchronization of fiber activations (τ). In all panels, results are compared across two myelinated fibers (4 and 8 μm) and three unmyelinated fibers (0.5, 1, and 1.5 μm). Recordings are monopolar in (E), while tripolar elsewhere.

In contrast to intraneural electrodes, cuff recordings have amplitudes mildly affected by the fiber position and well distinct across fiber types (Figure 6A). In addition, the degree of amplitude separation between unmyelinated and myelinated fibers is much more pronounced: for instance, comparing a 4 μm myelinated fiber with a 1 μm unmyelinated fiber, the separation results about two orders of magnitude with the cuff (Figure 6A), while it is just 5-fold with the TIME (Figure 5A) and 10-fold with the μNG (Figure 5B) at an intrafascicular fiber-to-electrode distance of 100 μm. The amplitudes of the SUAPs transduced by the cuff result particularly small for both myelinated and unmyelinated fibers, with amplitudes above 10 μV only associated with Aα fibers (> 12 μm, see Figure 3D, inset iii). This is not the result of critical modeling choices, as changing the thickness of the perineurium (Figure 6B) or the coverage of the active sites (Figure 6C) has a relatively small impact on the results. In stark contrast, wrapping the cuff around smaller peripheral nerves (0.5–1 mm diameter) dramatically increases the recording amplitude (Figure 6D). Designing longer cuffs may introduce some signal amplification, but this trend saturates at a few millimeters, especially for small-diameter fibers (Figure 6D). We thus sought to test whether larger signal amplitudes may result from compound action potentials, namely from the quasi-synchronous activation of fibers (Figure 6E). An exponential distribution was used to sample the activations of fibers, using the mean τ to control their degree of synchronization. We observed that for τ = 1 ms, a CAP peak-to-peak amplitude greater than 15 μV is induced by ten 8-μm myelinated fibers and by twenty 4-μm myelinated fibers. Increasing the number of active fibers further increases the CAP amplitude, for instance to more than 40 μV for 8-μm myelinated fibers.

### Electrode encapsulation may lead to the disappearance of the near-field regime

**Figure 7:**
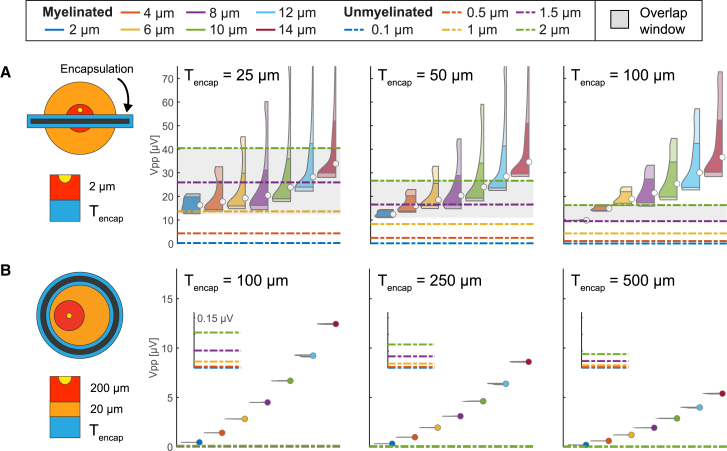
Effect of encapsulation tissue of implantable electrodes on the amplitude of SUAPs The longitudinal distributions of peak-to-peak amplitudes of SUAPs are shown for several fiber types in the presence of an encapsulation tissue surrounding the electrode. (A) Intrafascicular TIME evaluated with encapsulation thicknesses of 25, 50, and 100 μm. (B) Cuff evaluated with encapsulation thicknesses of 100, 250, and 500 μm. In each inset, the shaded area corresponds to the range of amplitudes shared by myelinated and unmyelinated fibers. Data are represented as violin plots for myelinated fibers and lines for unmyelinated fibers.

Encapsulation tissue is known to grow between electrodes and nerve tissues during chronic implantations, with thicknesses ranging from tens of micrometers for TIMEs^54^ up to 500 μm for cuffs.^55^ The presence of an encapsulation tissue around the TIME lowers the amplitude of SUAPs, increases the amplitude separation between SUAPs of different fiber types, and narrows the overlap window (Figure 7A). All these effects are proportional to the thickness of the encapsulation and qualitatively resemble those obtained by displacing the electrode from a given fiber (Figure 3, Figure 4D and 4D, inset i), as the encapsulation tissue effectively increases the distance between the fibers and the recording sites. Thus, thick encapsulation may cause intraneural electrodes to operate exclusively in the far-field regime (Figure 2C). The encapsulation tissue between the cuff and the nerve also reduces the amplitude of SUAPs (Figure 7B). This reduction in amplitude increases with the encapsulation thickness and is comparable to that observed when changing the nerve diameter (Figure 6D).

## Discussion

In the present work, we employed computational models of peripheral nerve recording to test whether the capability to record unmyelinated fiber activity in human-sized peripheral nerves is a unique feature of μNG needles or it is also shared by implantable electrodes. To this aim, we employed hybrid models to characterize the amplitude of the recording signal acquired from unmyelinated and myelinated fibers with TIME and μNG electrodes, in case of intrafascicular or extrafascicular insertion, and with a commercial cuff. Our analyses expand previous *in-silico* accounts on intraneural recordings focusing only on myelinated activity,^40^^,^^43^^,^^44^^,^^45^ and complement previous studies on extraneural recordings^41^^,^^42^ by considering a translationally relevant target as the human vagus nerve.

When the electrodes were located intrafascicularly and close to the target fiber (near-field region in Figure 2C), unmyelinated fibers displayed SUAP amplitudes comparable to or even higher than those recorded along large portions of the internodes in myelinated fibers (overlap window in Figure 3D). This effect was especially pronounced for unmyelinated fiber diameters larger than 0.5 μm, typical of autonomic nerves.^51^ The presence of the overlap window for both TIME and μNG electrodes suggests that the TIME can achieve transduction capabilities akin to the μNG needle, provided it is operated under similar conditions (i.e., relative electrode-fiber position). We explain the existence of the overlap window noting that membrane currents are uniform along unmyelinated fibers, but vary strongly when following the path of myelinated fibers, with higher currents corresponding to Ranvier nodes and significantly lower currents close to internodes (Figure S5; Table S2). As a result, when recording electrodes are close to a myelinated fiber but sufficiently far from Ranvier nodes, it may happen that they “collect” overall currents lower than in the case of an unmyelinated fiber, even if such currents are much larger close to Ranvier nodes. When the electrode is moved farther away from the fiber (far-field region in Figure 2C), multiple Ranvier nodes enter into the “field of view” of the electrode (Figures S6A and S6B), causing myelinated SUAP amplitude to decay much slower than for unmyelinated fibers, accompanied by a weaker longitudinal dependence, and a narrower overlap window (Figure 4D).

Comparing the TIME with the μNG, we found SUAPs with larger amplitudes and also a slower onset of fiber-wise amplitude separation (Figures 5A and 5B). This stems from the planar geometry of the TIME, which better confines the extracellular currents induced by the fiber, thereby ensuring larger and slower decaying *trans*-resistance values (Figures S6A and S6B). Consequently, for a given placement of fibers with respect to the electrode, unmyelinated fibers are transduced in a larger signal by the TIME compared to μNG. We note that this is expected to translate also in a more favorable signal-to-noise ratio for the TIME, since the amplitude of thermal noise is proportional to the square root of the electrode-electrolyte interface impedance,^56^ and *in vivo* impedances reach about 100 kΩ for TIME electrodes^57^ while up to 500 kΩ for μNG electrodes.^26^

Moving intraneural electrodes to an extrafascicular insertion resulted in attenuated SUAP amplitudes and a quicker onset of amplitude separation (Figures 5C and 5D), due to reduced electrode-fiber coupling caused by the shielding effect of the perineurium and the lower conductivity of the epineurium tissue (Figures S6C and S6D). As a result, unmyelinated SUAPs were confined to peak-to-peak amplitudes of less than a few μV and are likely even smaller in practice, as fibers are typically located deeper within fascicles than assumed in our analysis (10 μm, as the minimum perineurium thickness of the human vagus nerve^46^). This suggests that distinguishing unmyelinated fibers from the experimental noise floor would be challenging when intraneural electrodes fail to penetrate fascicles. Similar conclusions may be drawn for the cuff, where unmyelinated SUAPs resulted tens to hundreds fold smaller than their myelinated counterparts (Figure 3D), with such amplitude separation showing weakly dependence upon the fiber-to-electrode distance (Figure 6A). Indeed, the cuff imposes a longitudinal propagation of currents within the surrounded nerve trunk, leading to a lack of radial dependence in the lead-field matrix in the endoneurium and epineurium (Figure 1C). Consequently, the placement of fascicles and fibers has only a mild influence on *trans*-resistance values (Figures S6E and S6F) and SUAPs (Figure S7). In addition, no longitudinal dependence was observed for myelinated SUAPs due to the relatively large size of the electrode, concurrently collecting the electrogenic activity of several internode segments (Figures S6E and S6F). The relatively small amplitude of SUAPs, even of myelinated fibers, suggests that compound activity from multiple fibers is essential for transducing signals above the noise floor (Figure 6E). This is indirectly supported by electrically evoked compound action potentials (ECAPs) in the pig vagus nerve,^25^^,^^51^ which display distinct peaks corresponding to the synchronous activation of large-myelinated and small-myelinated fibers.

Effective recording of unmyelinated fibers from human-sized peripheral nerves is relevant for translational purposes, as their discharges encode physiological-relevant information that may be exploited in bioelectronic therapies and neuroprostheses. Our work provides *in-silico* evidence that this ability is not inherent to specific physical properties of μNG needles but other intraneural electrodes, such as the TIME, emerge as promising alternatives for chronic application, offering even more favorable signal-to-noise ratio. In contrast, cuff electrodes may not be well-suited to this aim, as the size of human peripheral nerves fundamentally limits the amplitude of the transduced signals. The TIME electrode represents an ideal candidate for this translational purpose, as it has already been tested chronically in human amputees up to several months,^58^ without reported safety-related issues, and biocompatibility confirmed by immunohistochemical animal studies.^54^ In addition, despite recordings have been performed only in limited time frames thus far,^16^ the TIME stability has been extensively proven through the longitudinal tracking of evoked sensory percepts.^59^ Notwithstanding, we envision several challenges that must be addressed to maintain TIME recordings of unmyelinated fibers above the noise floor. These include limiting the thickness of encapsulation tissue (Figure 7) and developing techniques to reject artifacts from non-neural sources, similar to what is achieved through tripolar configurations in cuffs.^49^ We highlight that clinically relevant alternatives to intraneural electrodes exist for effectively transducing neural activity, such as regenerative interfaces.^60^ However, while these interfaces are well-suited for capturing neural motor commands by leveraging the natural signal amplification of muscles, their potential for recording unmyelinated fiber activity has yet to be explored.

Building upon our results, we suggest that the missing evidence of recorded unmyelinated activity with TIME electrodes from human-sized peripheral nerves may be linked to systematic differences in the employed experimental setups compared to microneurographic studies. For instance, intrafascicular insertion is ensured in uNG studies through real-time monitoring of the recorded nerve activity until the onset of a large signal-to-noise ratio.^12^ Implantation of the TIME electrode is instead blind, with recording sessions performed after the surgery.^16^ As a result, TIMEs typically fail to penetrate the tight perineurial sheath of fascicles, as confirmed by post-explant histological studies in large animals.^27^^,^^30^ In addition, while uNG studies are performed in awake subjects, TIME recordings from autonomic nerves have thus far been conducted under anesthesia,^14^^,^^27^ which is known to alter nerve electrogenic activity.^28^^,^^29^ Although recordings have been performed in awake subjects from somatic nerves,^16^ no experimental protocols specifically designed to excite unmyelinated activity have been implemented in these settings. We thus motivate future experiments aimed at minimizing these discrepancies, such as surgical procedures to guide electrode insertion within fascicles and chronic studies enabling recordings of unmodified autonomic activity. This is crucial for validating the potential of the TIME electrode in transducing unmyelinated fibers, and, consequently, in utilizing their real-time activity for neuroengineering applications.

The small amplitude recordings observed for the cuff challenge the feasibility of translating unmyelinated fiber recordings achieved with such electrodes in small-sized animals^24^ to humans. Indeed, the size of the nerve enclosed by the cuff emerges as a critical factor in determining the transduced signal strength, with much larger SUAPs predicted in small-diameter nerves (e.g., 0.5 mm, see Figure 6D). This is in agreement with predictions of previous models of cuff recordings from rat peripheral nerves.^41^ Although the presence of compound activity might generate recording signals above the noise floor for unmyelinated fibers (Figure 6E), this possibility remains elusive. Indeed, while presence of synchronous unmyelinated activity has been reported in ECAPs from the human vagus nerve,^61^ recent studies in pigs have challenged this interpretation, identifying muscle electrical contamination as the source of these signal components.^62^ Future experiments may shed better light on the possibility of recording both single-unit and compound activity of unmyelinated fibers with cuff electrodes.

### Limitations of the study

We remark that our characterization of peripheral recording amplitudes is valid under certain assumptions. First, we employ the quasistatic approximation^63^ to solve the volume conductor problem and build the lead-field matrix, neglecting capacitive and dispersive effects in the tissues, as well as an explicit representation of the impedance at the electrode-tissue interface. While these assumptions are justifiable for predicting recording waveforms, they prevent to simulate the impedance seen by the electrode and, consequently, thermal noise.^56^ Incorporating this feature would enable direct comparisons between signal recordings and noise floor levels, which is crucial for evaluating the potential of intraneural electrodes, given their high impedance values observed *in vivo*.^26^^,^^57^ Second, we consider an extracellular coupling between electrodes and fibers: this excludes cases of intracellular coupling (e.g., μNG needles piercing a nerve fiber^26^), which would require an explicit treatment of fiber geometry in the volume conduction problem,^64^ beyond the adopted hybrid modeling formalism. Third, simplified nerve geometries and fiber paths are modeled through extrusion along the longitudinal direction. While more realistic nerve are known to affect the simulated recording signal,^41^ evaluating the extent to which these changes may impact the main observations of the work is left for future investigations. Finally, using a tripolar configuration for the cuff, while monopolar configurations for TIME and μNG, is not a critical choice, as SUAPs with similar amplitudes are observed with the cuff in a monopolar configuration, despite the longer duration (Figure S8).

## Resource availability

### Lead contact

Further information and requests should be directed to the lead contact, Simone Romeni (romeni.simone@hsr.it).

### Materials availability

This study did not generate new unique reagents.

### Data and code availability

• All data reported in this paper will be shared by the lead contact upon request. However, since all data have been generated *in silico*, they can be fully reproduced using the provided code (see below).
• All original code has been deposited on GitHub and is publicly available at https://github.com/claudioverardo/nerve_rec_characterization as of the date of publication.
• Any additional information required to reanalyze the data reported in this paper is available from the lead contact upon request.

## Acknowledgments

This study was partly funded by the NIH 10.13039/501100019550SPARC funding program—REVEAL project (U54AT012307), and 10.13039/100009152Bertarelli Foundation.

## Author contributions

Conceptualization: C.V., S.R., and S.M.; methodology: C.V. and S.R.; software: C.V.; formal analysis: C.V.; investigation: C.V.; resources: S.M.; data curation: C.V.; writing – original draft: C.V., S.R., and S.M.; writing – review and editing: C.V., S.R., and S.M.; visualization: C.V.; supervision: S.R. and S.M.;

## Declaration of interests

The authors declare no competing interests.

## STAR★Methods

### Key resources table

**Table:** 

REAGENT or RESOURCE	SOURCE	IDENTIFIER
**Software and algorithms**		

Simulation and characterization of peripheral nerve recordings	This paper	https://github.com/claudioverardo/nerve_rec_characterization
COMSOL Multiphysics 6.2	COMSOL Inc.	https://www.comsol.com/
NEURON 8.2.2	Duke, Yale, and the BlueBrain Project	https://www.neuron.yale.edu
MATLAB R2023a	MathWorks, Inc.	https://mathworks.com/
Python 3.10.11	Python Software Foundation	https://www.python.org/

### Method details

The modeling workflow is illustrated in Figure 1 and summarized in the flowchart in Figure S9.

#### Nerve and electrodes geometry

We modeled the nerve using a simplified cylindrical geometry, with 2 mm diameter and 4 cm length (Figure 1A). A cylindrical fascicle with 400 μm diameter was also included, positioned at the center of the cross-section, if not otherwise stated. The size of the nerve and the fascicle are based on morphological distributions for the human cervical vagus nerve.^46^ Fibers were positioned in the nerve cross-section and discretized along the longitudinal direction in compartments (see Section “fiber excitation templates”). The central locations of the fiber compartments were thus co-registered with the nerve geometry, possibly accounting for a longitudinal shift, to allow for the simulation of peripheral recordings (see Section “lead-field matrix of recording sites”). A cylindrical saline bath with 10 mm diameter and same length of the nerve surrounded all the geometry to mimic the intraoperative environment.

We considered models of TIME, μNG, and cuff electrodes for implantation in the nerve (Figure 1B). The TIME electrode replicates the design presented in,^65^^,^^66^ which adapts the original specifications^67^ for implantation in the cervical vagus nerve of swines. The polyimide insulator has a thickness of 19 μm, a width of 750 μm, and a length of 4 mm. A single active site is embedded in the polyimide at the center of one side of the electrode and has a circular cross-section with a diameter of 80 μm. The μNG electrode reflects the typical geometrical parameters of needle electrodes used in microneurographic experiments.^26^ It has an insulated shaft with a circular cross-section of 200 μm diameter and a length of 5 mm. A tip is formed at one side by tapering the shaft with an 80° slope and a final rounding of 5 μm diameter. The active site corresponds to the last 30 μm of the tip, while the rest of the tip is insulated. The cuff electrode is based on the design of commercial cuffs from CorTec GmbH (Freiburg, Germany, https://www.cortec-neuro.com/). The silicone insulator is modeled as a hollow cylinder with an internal radius equal to the nerve, length 15 mm, and thickness 1 mm. Three ring active sites are modeled with 1 mm width and 4 mm center-to-center longitudinal pitch.

The electrodes were implanted at half of the modeled nerve segment. In detail, the TIME and μNG electrodes were placed perpendicularly to the longitudinal direction of the nerve, with the active site either inside the fascicle (intrafascicular insertion) or outside the fascicle (extrafascicular insertion). The cuff’s inner surface was in direct contact with the outer boundary of the nerve.

#### Lead-field matrix of recording sites

We employed finite-element methods (FEM) to solve the volume conductor problem, i.e., to compute the extracellular potential in the modeled geometry upon injection of a given combination of currents from the active sites. This was done under the quasistatic approximation by solving the Laplace equation ∇·(σ∇V)=0, where V represents the extracellular potential and σ denotes the spatially dependent conductivity.^31^ The injected currents were applied by setting the total current flux at the active site boundaries, while grounding the outermost surfaces of the saline bath. The conductivities were assigned according to literature^31^: 0.083 S/m and 0.571 S/m for the radial and longitudinal directions of the endoneurium, 0.083 S/m for the epineurium, 10^10^ S/m for the active sites, 10^−10^ S/m for the electrode insulator, 0.159 S/m for the electrode encapsulation tissue, and 2 S/m for the saline bath. The perineurium sheath was regarded as a boundary impedance assuming a thin-film approximation, with conductivity set to 0.0009 S/m^31^ and thickness determined by the fascicle diameter according to fit on the human vagus nerve.^46^ The volume conductor problem was solved using Comsol Multiphysics.

Following the reciprocity theorem,^32^ we used the solution of the volume conductor problem to compute the lead field matrix (LFM) for each active site, i.e., the trans-resistance values between a recording site and the fiber compartments. The trans-resistance $R_{ij}$ defines the voltage read-out $V_{i}$ at the i-th active site generated by a current source $I_{j}$ at the j-th fiber compartment as $V_{i}=R_{ij}\;I_{j}$. The trans-resistances $R_{ij}$ for the i-th active site were obtained by computing the extracellular potential induced by a 1 μA current from the active site, dividing it by the injected current, and sampling the results at the center of the fiber compartments. We note that the reciprocity formalism allows for a massive reduction of computational time to obtain the LFMs, since a FEM simulation is needed only for each recording site and not for all the fiber compartments.^34^ We also point out that the fibers do not influence the volume conductor model and its solution.

#### Fiber excitation templates

The electrogenic activity of fibers was simulated with multicompartmental axon models in Neuron,^68^ through its Python interface. The MRG model^47^ was used for myelinated fibers, while the Sundt model^48^ was used for unmyelinated fibers. They were implemented according to the HOC code available in ASCENT.^69^ Myelinated fibers were spatially discretized in compartments representing the Ranvier nodes, paranodal segments (MYSA), juxtaparanodal segments (FLUT), and internodal segments (STIN), whose ultrastructural parameters were set according to the fiber diameter^69^ (Table S2). The region between two consecutive Ranvier nodes was composed of 2 MYSA, 2 FLUT, and 6 STIN compartments Figure 2A), and was globally referred to as internodal region in the main text. The spatial discretization step of unmyelinated fibers was 50/6 μm.^69^

For each fiber type, we pre-computed the spatiotemporal evolution of the transmembrane current during the propagation of an action potential, also referred to as fiber excitation template. To reduce the amount of data to be stored,^34^ we saved the transmembrane current only at one internode segment for myelinated fibers and to one compartment for unmyelinated fibers. When needed, the excitation template of the full fiber was reconstructed by time-shifting the stored transmembrane currents to the other compartments according to their longitudinal positions and the fiber conduction velocity.^41^^,^^42^ More in detail, the excitation template of transmembrane currents for each myelinated fiber was obtained by instantiating a fiber with 360 internodes and applying a step current of 0.5 nA for 1 ms as intracellular stimulus at the first node of Ranvier. The transmembrane currents of the internode compartments (Ranvier node, MYSA #1, FLUT #1, STIN #1, STIN #2, STIN #3, STIN #4, STIN #5, #STIN #6, FLUT #2, MYSA #2) were stored starting from the 100-th Ranvier node, to minimize the stimulation artifact. The excitation template of each unmyelinated fiber was obtained with the same stimulus, but instantiating 1251 fiber nodes and storing the transmembrane currents at the 200-th compartment. A time discretization step of 2.5 μs was used for myelinated fibers, while of 10 μs for unmyelinated fibers. Alongside the excitation templates, the conduction velocity of the elicited action potential was estimated.

#### Simulation of peripheral recordings

For a given combination of electrode geometry, electrode insertion, and fibers, recording signals were generated according to the hybrid modeling formalism introduced in.^32^ First, the LFM at a given recording site was computed (Figure 1C). Second, the LFM along each fiber was multiplied by its excitation template to produce its single-unit action potential (SUAP), i.e., the recording waveform associated to the propagation of a single action potential through the fiber (Figure 1D). The process was repeated for each active site, thereby obtaining the monopolar SUAPs associated to the electrode. Multi-unit recordings (MUAP) were obtained by convolving the SUAP of each fiber with its train of activations and then summing the results across fibers (Figure 1E). For TIME and μNG electrodes, we considered only monopolar recordings. For the cuff electrode, if not stated otherwise, we simulated recordings in tripolar configuration by subtracting from the monopolar recordings of the middle active site the average of the monopolar recordings of the lateral pair of active sites^35^ (Figure 1F). To minimize the numerical instabilities in the unmyelinated SUAPs of cuff electrodes, we decreased the discretization length in the LFM of unmyelinated fibers by a factor of 10 and interpolated the excitation templates accordingly. The generation of SUAPs and MUAPs was done in Matlab.

#### Spatial maps and longitudinal distributions of SUAP amplitudes

For selected combinations of electrode insertion, electrode geometry, and fiber type, we built the spatial map of SUAP amplitudes (Figure 2). Specifically, we plotted the peak-to-peak amplitudes of the SUAPs obtained by varying the fiber-to-electrode distance and the placement of the electrode along the fiber. The fiber was placed at the center of the fascicle for intrafascicular insertions and the cuff, while at 10 μm from the fascicle border for extrafascicular insertions (see the main text for a thorough motivation of these choices). The electrode-to-fiber distance was changed by a radial displacement of the electrode in the case of intraneural electrodes (2 μm steps), while by moving the fascicle in the case of the cuff (20 μm steps). The position of the electrode along the fiber was modified by adding a longitudinal shift to the position of the fiber compartments with respect to the nerve geometry. To characterize such dependence in myelinated fibers, it is sufficient to consider shifts spanning a single internodal length due to the periodic nature of the problem. Therefore, we assumed a discretization step of 5 μm, starting from two consecutive Ranvier nodes and moving towards the midpoint of the enclosed internode. Although unmyelinated fibers do not exhibit such longitudinal dependence, we used the same visualization approach as for myelinated fibers but evaluating the SUAPs without any longitudinal shift. The same procedure was followed to compute the longitudinal distributions of SUAP peak-to-peak amplitudes at fixed distances between electrodes and fibers (e.g., in Figures 3D and 4D).

#### Compound action potential amplitudes

In Figure 6E, we simulated compound action potentials (CAPs) resulting from the summation of the recording signals from quasi-synchronous active fibers. Specifically, N fibers were randomly placed in the nerve fascicle, and each was assigned with an activation timing t_k_, sampled from an exponential distribution with mean value τ. Such fiber activations were assumed to occur at the beginning of the modeled nerve geometry. Simulations were performed for different fiber types, across different values of N and τ, and the resulting peak-to-peak amplitudes of the CAPs were computed. We note that CAPs are a special case of MUAP activity, whose simulation details are reported in Section “simulation of peripheral recordings”.

## References

[1] Zanos S. (2019). Closed-loop neuromodulation in physiological and translational research. Cold Spring Harb. Perspect. Med..

[2] Raspopovic S., Cimolato A., Panarese A., Vallone F., del Valle J., Micera S., Navarro X. (2020). Neural signal recording and processing in somatic neuroprosthetic applications. A review. J. Neurosci. Methods.

[3] Valle G., Mazzoni A., Iberite F., D’Anna E., Strauss I., Granata G., Controzzi M., Clemente F., Rognini G., Cipriani C. (2018). Biomimetic Intraneural Sensory Feedback Enhances Sensation Naturalness, Tactile Sensitivity, and Manual Dexterity in a Bidirectional Prosthesis. Neuron.

[4] Zanos T.P., Silverman H.A., Levy T., Tsaava T., Battinelli E., Lorraine P.W., Ashe J.M., Chavan S.S., Tracey K.J., Bouton C.E. (2018). Identification of cytokine-specific sensory neural signals by decoding murine vagus nerve activity. Proc. Natl. Acad. Sci. USA.

[5] Steinberg B.E., Silverman H.A., Robbiati S., Gunasekaran M.K., Tsaava T., Battinelli E., Stiegler A., Bouton C.E., Chavan S.S., Tracey K.J., Huerta P.T. (2016). Cytokine-specific Neurograms in the Sensory Vagus Nerve. Bioelectron. Med..

[6] Jiman A.A., Ratze D.C., Welle E.J., Patel P.R., Richie J.M., Bottorff E.C., Seymour J.P., Chestek C.A., Bruns T.M. (2020). Multi-channel intraneural vagus nerve recordings with a novel high-density carbon fiber microelectrode array. Sci. Rep..

[7] Güemes Gonzalez A., Carnicer-Lombarte A., Hilton S., Malliaras G.G. (2023). A multivariate physiological model of vagus nerve signalling during metabolic challenges in anaesthetised rats for diabetes treatment. J. Neural. Eng..

[8] Pollina L., Vallone F., Ottaviani M.M., Strauss I., Carlucci L., Recchia F.A., Micera S., Moccia S. (2022). A lightweight learning-based decoding algorithm for intraneural vagus nerve activity classification in pigs. J. Neural. Eng..

[9] Sevcencu C., Nielsen T.N., Struijk J.J. (2018). An Intraneural Electrode for Bioelectronic Medicines for Treatment of Hypertension. Neuromodulation.

[10] Sevcencu C., Nielsen T.N., Kjærgaard B., Struijk J.J. (2018). A Respiratory Marker Derived From Left Vagus Nerve Signals Recorded With Implantable Cuff Electrodes. Neuromodulation.

[11] Metcalfe B.W., Nielsen T.N., Donaldson N. de N., Hunter A.J., Taylor J.T. (2018). First demonstration of velocity selective recording from the pig vagus using a nerve cuff shows respiration afferents. Biomed. Eng. Lett..

[12] Patros M., Ottaviani M.M., Wright L., Dawood T., Macefield V.G. (2022). Quantification of cardiac and respiratory modulation of axonal activity in the human vagus nerve. J. Physiol..

[13] Farmer D.G.S., Patros M., Ottaviani M.M., Dawood T., Kumric M., Bozic J., Badour M.I., Bain A.R., Barak O.F., Dujic Z. (2024). Firing properties of single axons with cardiac rhythmicity in the human cervical vagus nerve. J. Physiol..

[14] Giannotti A., Lo Vecchio S., Musco S., Pollina L., Vallone F., Strauss I., Paggi V., Bernini F., Gabisonia K., Carlucci L. (2023). Decoding bladder state from pudendal intraneural signals in pigs. APL Bioeng..

[15] Mathews K.S., Wark H.A.C., Warren D.J., Christensen M.B., Nolta N.F., Cartwright P.C., Normann R.A. (2014). Acute monitoring of genitourinary function using intrafascicular electrodes: Selective pudendal nerve activity corresponding to bladder filling, bladder fullness, and genital stimulation. Urology.

[16] Cracchiolo M., Valle G., Petrini F., Strauss I., Granata G., Stieglitz T., Rossini P.M., Raspopovic S., Mazzoni A., Micera S. (2020). Decoding of grasping tasks from intraneural recordings in trans-radial amputee. J. Neural. Eng..

[17] Wendelken S., Page D.M., Davis T., Wark H.A.C., Kluger D.T., Duncan C., Warren D.J., Hutchinson D.T., Clark G.A. (2017). Restoration of motor control and proprioceptive and cutaneous sensation in humans with prior upper-limb amputation via multiple Utah Slanted Electrode Arrays (USEAs) implanted in residual peripheral arm nerves. J. NeuroEng. Rehabil..

[18] Nguyen A.T., Xu J., Jiang M., Luu D.K., Wu T., Tam W.K., Zhao W., Drealan M.W., Overstreet C.K., Zhao Q. (2020). A bioelectric neural interface towards intuitive prosthetic control for amputees. J. Neural. Eng..

[19] Haugland M., Lickel A., Haase J., Sinkjær T. (1999). Control of FES thumb force using slip information obtained from the cutaneous electroneurogram in quadriplegic man. IEEE Trans. Rehabil. Eng..

[20] Tubbs R.S., Rizk E., Shoja M.M., Loukas M., Barbaro N., Spinner R.J. (2015). Nerves and Nerve Injuries - Vol 1: History, Embryology, Anatomy, Imaging, and Diagnostics.

[21] Wehrwein E.A., Orer H.S., Barman S.M. (2016). Overview of the Anatomy, Physiology, and Pharmacology of the Autonomic Nervous System. Compr. Physiol..

[22] Ottaviani M.M., Macefield V.G. (2022). Structure and Functions of the Vagus Nerve in Mammals. Compr. Physiol..

[23] McGlone F., Reilly D. (2010). The cutaneous sensory system. Neurosci. Biobehav. Rev..

[24] Carnicer-Lombarte A., Boys A.J., Güemes A., Gurke J., Velasco-Bosom S., Hilton S., Barone D.G., Malliaras G.G. (2024). Ultraconformable cuff implants for long-term bidirectional interfacing of peripheral nerves at sub-nerve resolutions. Nat. Commun..

[25] Verma N., Knudsen B., Gholston A., Skubal A., Blanz S., Settell M., Frank J., Trevathan J., Ludwig K. (2023). Microneurography as a minimally invasive method to assess target engagement during neuromodulation. J. Neural. Eng..

[26] Vallbo Å.B. (2018). Microneurography: How it started and how it works. J. Neurophysiol..

[27] Vallone F., Ottaviani M.M., Dedola F., Cutrone A., Romeni S., Panarese A.M., Bernini F., Cracchiolo M., Strauss I., Gabisonia K. (2021). Simultaneous decoding of cardiovascular and respiratory functional changes from pig intraneural vagus nerve signals. J. Neural. Eng..

[28] Silverman H.A., Stiegler A., Tsaava T., Newman J., Steinberg B.E., Masi E.B., Robbiati S., Bouton C., Huerta P.T., Chavan S.S., Tracey K.J. (2018). Standardization of methods to record Vagus nerve activity in mice. Bioelectron. Med..

[29] Falcone J.D., Liu T., Goldman L., David D P., Rieth L., Bouton C.E., Straka M., Sohal H.S. (2020). A novel microwire interface for small diameter peripheral nerves in a chronic, awake murine model. J. Neural. Eng..

[30] Kundu A., Harreby K.R., Yoshida K., Boretius T., Stieglitz T., Jensen W. (2014). Stimulation selectivity of the “thin-film longitudinal intrafascicular electrode” (tfLIFE) and the “transverse intrafascicular multi-channel electrode” (TIME) in the large nerve animal model. IEEE Trans. Neural Syst. Rehabil. Eng..

[31] Romeni S., Valle G., Mazzoni A., Micera S. (2020). Tutorial: a computational framework for the design and optimization of peripheral neural interfaces. Nat. Protoc..

[32] Struijk J.J. (1997). The extracellular potential of a myelinated nerve fiber in an unbounded medium and in nerve cuff models. Biophys. J..

[33] Andreasen L.N., Struijk J.J., Lawrence S. (2000). Measurement of the performance of nerve cuff electrodes for recording. Med. Biol. Eng. Comput..

[34] Peña E., Pelot N.A., Grill W.M. (2024). Computational models of compound nerve action potentials: Efficient filter-based methods to quantify effects of tissue conductivities, conduction distance, and nerve fiber parameters. PLoS Comput. Biol..

[35] Perez-Orive J., Durand D.M. (2000). Modeling study of peripheral nerve recording selectivity. IEEE Trans. Rehabil. Eng..

[36] Yoo P.B., Durand D.M. (2005). Selective recording of the canine hypoglossal nerve using a multicontact flat interface nerve electrode. IEEE Trans. Biomed. Eng..

[37] Sabetian P., Yoo P.B. (2020). Feasibility of differentially measuring afferent and efferent neural activity with a single nerve cuff electrode. J. Neural. Eng..

[38] Zariffa J., Popovic M.R. (2009). Localization of active pathways in peripheral nerves: A simulation study. IEEE Trans. Neural Syst. Rehabil. Eng..

[39] Garai P., Koh R.G.L., Schuettler M., Stieglitz T., Zariffa J. (2017). Influence of Anatomical Detail and Tissue Conductivity Variations in Simulations of Multi-Contact Nerve Cuff Recordings. IEEE Trans. Neural Syst. Rehabil. Eng..

[40] Pitzus A., Romeni S., Vallone F., Micera S. (2022). A method to establish functional vagus nerve topography from electro-neurographic spontaneous activity. Patterns.

[41] Lubba C.H., Le Guen Y., Jarvis S., Jones N.S., Cork S.C., Eftekhar A., Schultz S.R. (2019). PyPNS: Multiscale Simulation of a Peripheral Nerve in Python. Neuroinformatics.

[42] Eiber C.D., Payne S.C., Biscola N.P., Havton L.A., Keast J.R., Osborne P.B., Fallon J.B. (2021). Computational modelling of nerve stimulation and recording with peripheral visceral neural interfaces. J. Neural. Eng..

[43] Jehenne B., Raspopovic S., Capogrosso M., Arleo A., Micera S. (2015). International IEEE/EMBS Conference on Neural Engineering.

[44] Qiao S., Yoshida K. (2013). Influence of unit distance and conduction velocity on the spectra of extracellular action potentials recorded with intrafascicular electrodes. Med. Eng. Phys..

[45] Petrini F.M., Mazzoni A., Rigosa J., Giambattistelli F., Granata G., Barra B., Pampaloni A., Guglielmelli E., Zollo L., Capogrosso M. (2019). Microneurography as a tool to develop decoding algorithms for peripheral neuro-controlled hand prostheses. Biomed. Eng. Online.

[46] Pelot N.A., Goldhagen G.B., Cariello J.E., Musselman E.D., Clissold K.A., Ezzell J.A., Grill W.M. (2020). Quantified Morphology of the Cervical and Subdiaphragmatic Vagus Nerves of Human, Pig, and Rat. Front. Neurosci..

[47] McIntyre C.C., Richardson A.G., Grill W.M. (2002). Modeling the excitability of mammalian nerve fibers: Influence of afterpotentials on the recovery cycle. J. Neurophysiol..

[48] Sundt D., Gamper N., Jaffe D.B. (2015). Spike propagation through the dorsal root ganglia in an unmyelinated sensory neuron: A modeling study. J. Neurophysiol..

[49] Stein R.B., Charles D., Davis L., Jhamandas J., Mannard A., Nichols T.R. (1975). Principles Underlying New Methods for Chronic Neural Recording. Can. J. Neurol. Sci..

[50] Gasser H.S. (1941). The classification of nerve fibers. Ohio J. Sci..

[51] Jayaprakash N., Song W., Toth V., Vardhan A., Levy T., Tomaio J., Qanud K., Mughrabi I., Chang Y.C., Rob M. (2023). Organ- and function-specific anatomical organization of vagal fibers supports fascicular vagus nerve stimulation. Brain Stimul..

[52] Plonsey R., Barr R.C. (2007).

[53] Grinberg Y., Schiefer M.A., Tyler D.J., Gustafson K.J. (2008). Fascicular perineurium thickness, size, and position affect model predictions of neural excitation. IEEE Trans. Neural Syst. Rehabil. Eng..

[54] Wurth S., Capogrosso M., Raspopovic S., Gandar J., Federici G., Kinany N., Cutrone A., Piersigilli A., Pavlova N., Guiet R. (2017). Long-term usability and bio-integration of polyimide-based intra-neural stimulating electrodes. Biomaterials.

[55] Grill W.M., Mortimer J.T. (2000). Neural and connective tissue response to long-term implantation of multiple contact nerve cuff electrodes. J. Biomed. Mater. Res..

[56] Boehler C., Carli S., Fadiga L., Stieglitz T., Asplund M. (2020). Tutorial: guidelines for standardized performance tests for electrodes intended for neural interfaces and bioelectronics. Nat. Protoc..

[57] Čvančara P., Valle G., Müller M., Bartels I., Guiho T., Hiairrassary A., Petrini F., Raspopovic S., Strauss I., Granata G. (2023). Bringing sensation to prosthetic hands—chronic assessment of implanted thin-film electrodes in humans. npj Flexible Electronics.

[58] Raspopovic S., Valle G., Petrini F.M. (2021). Sensory feedback for limb prostheses in amputees. Nat. Mater..

[59] Valle G., Aiello G., Ciotti F., Cvancara P., Martinovic T., Kravic T., Navarro X., Stieglitz T., Bumbasirevic M., Raspopovic S. (2022). Multifaceted understanding of human nerve implants to design optimized electrodes for bioelectronics. Biomaterials.

[60] Vu P.P., Vaskov A.K., Irwin Z.T., Henning P.T., Lueders D.R., Laidlaw A.T., Davis A.J., Nu C.S., Gates D.H., Gillespie R.B. (2020). A regenerative peripheral nerve interface allows real-time control of an artificial hand in upper limb amputees. Sci. Transl. Med..

[61] Evans M.S., Verma-Ahuja S., Naritoku D.K., Espinosa J.A. (2004). Intraoperative human vagus nerve compound action potentials. Acta Neurol. Scand..

[62] Nicolai E.N., Settell M.L., Knudsen B.E., McConico A.L., Gosink B.A., Trevathan J.K., Baumgart I.W., Ross E.K., Pelot N.A., Grill W.M. (2020). Sources of off-target effects of vagus nerve stimulation using the helical clinical lead in domestic pigs. J. Neural. Eng..

[63] Wang B., Peterchev A.V., Gaugain G., Ilmoniemi R.J., Grill W.M., Bikson M., Nikolayev D. (2024). Quasistatic approximation in neuromodulation. J. Neural. Eng..

[64] Agudelo-Toro A., Neef A. (2013). Computationally efficient simulation of electrical activity at cell membranes interacting with self-generated and externally imposed electric fields. J. Neural. Eng..

[65] Agnesi F., Zinno C., Strauss I., Dushpanova A., Casieri V., Bernini F., Terlizzi D., Gabisonia K., Paggi V., Lacour S.P. (2024). Cardiovascular Response to Intraneural Right Vagus Nerve Stimulation in Adult Minipig. Neuromodulation.

[66] Strauss I., Agnesi F., Zinno C., Giannotti A., Dushpanova A., Casieri V., Terlizzi D., Bernini F., Gabisonia K., Wu Y. (2023). Neural Stimulation Hardware for the Selective Intrafascicular Modulation of the Vagus Nerve. IEEE Trans. Neural Syst. Rehabil. Eng..

[67] Boretius T., Badia J., Pascual-Font A., Schuettler M., Navarro X., Yoshida K., Stieglitz T. (2010). A transverse intrafascicular multichannel electrode (TIME) to interface with the peripheral nerve. Biosens. Bioelectron..

[68] Carnevale N.T., Hines M.L. (2006).

[69] Musselman E.D., Cariello J.E., Grill W.M., Pelot N.A. (2021). ASCENT (Automated Simulations to Characterize Electrical Nerve Thresholds): A pipeline for sample-specific computational modeling of electrical stimulation of peripheral nerves. PLoS Comput. Biol..

