# Supplementary material for: Biophysical characterization of the recording of unmyelinated and myelinated fiber activity with peripheral interfaces
Source: iScience. 2025 Apr 22;28(5):112495. doi: 10.1016/j.isci.2025.112495 (PMC12127600; doi:10.1016/j.isci.2025.112495)
Supplement: Document S1. Figures S1–S9 and Tables S1 and S2 [file mmc1.pdf]

iScience, Volume *28*

Supplemental information

Biophysical characterization of the recording of unmyelinated and myelinated fiber activity with peripheral interfaces

Claudio Verardo, Simone Romeni, and Silvestro Micera

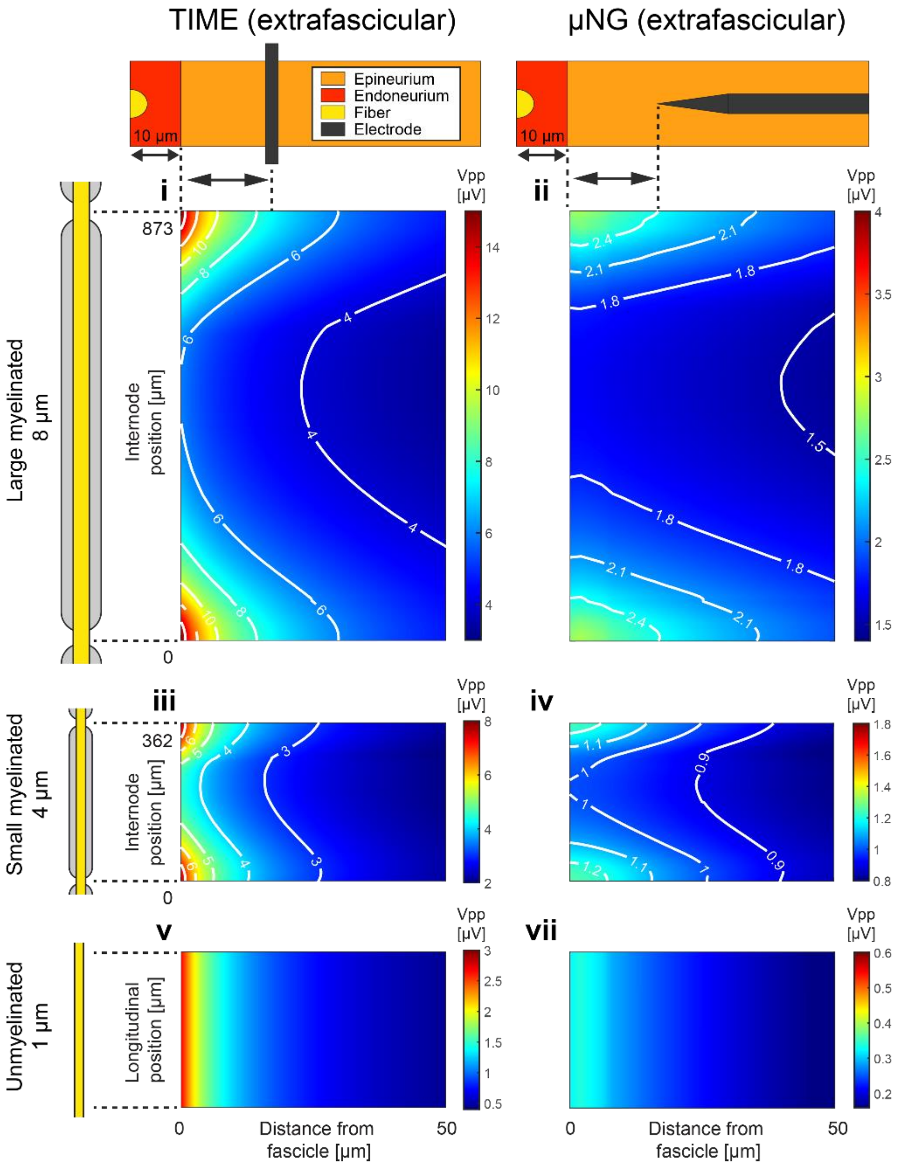

Figure S1. Dependence of SUAP amplitudes upon the electrode-fiber relative position (extrafascicular insertions), related to Figure 2. The spatial maps of SUAP amplitudes are shown for the case of extrafascicular insertion of TIME and µNG.

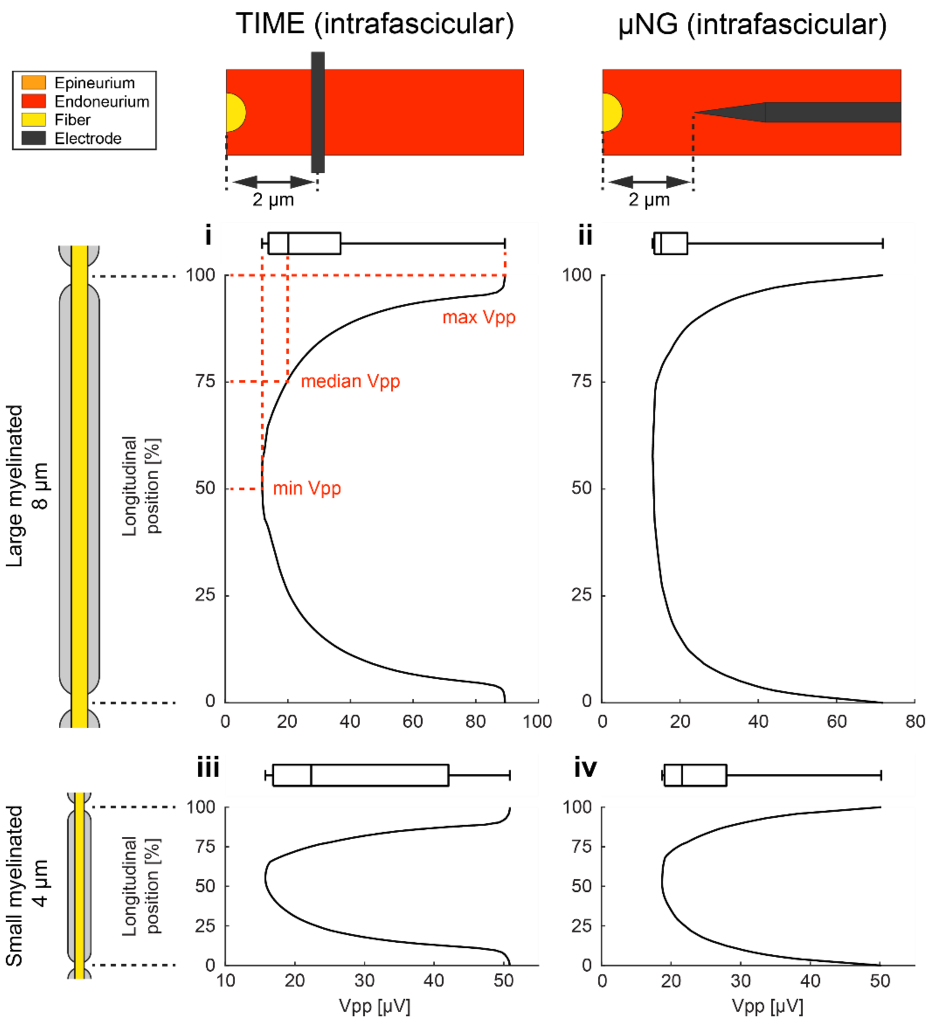

Figure S2. SUAP amplitudes recorded along myelinated fibers at fixed electrode-fiber distance, related to Figure 3. The insets (i-iv) consider different combinations of fiber type and electrode geometry and insertion. Rows refer to 8 µm-myelinated and 4 µm-myelinated fibers. Columns refer to intrafascicular TIME and intrafascicular µNG. A distance of 2 µm is assumed between electrodes and fibers. Each inset displays the distribution of peak-to-peak amplitudes of SUAPs (x axis) recorded along the internode (y axis), with boxplots denoting the main statistics of the distribution. Inset i highlights that the minimum, median, and maximum are found approximately at half of the internode, three quarters of the internode, and at the Ranvier node, respectively.

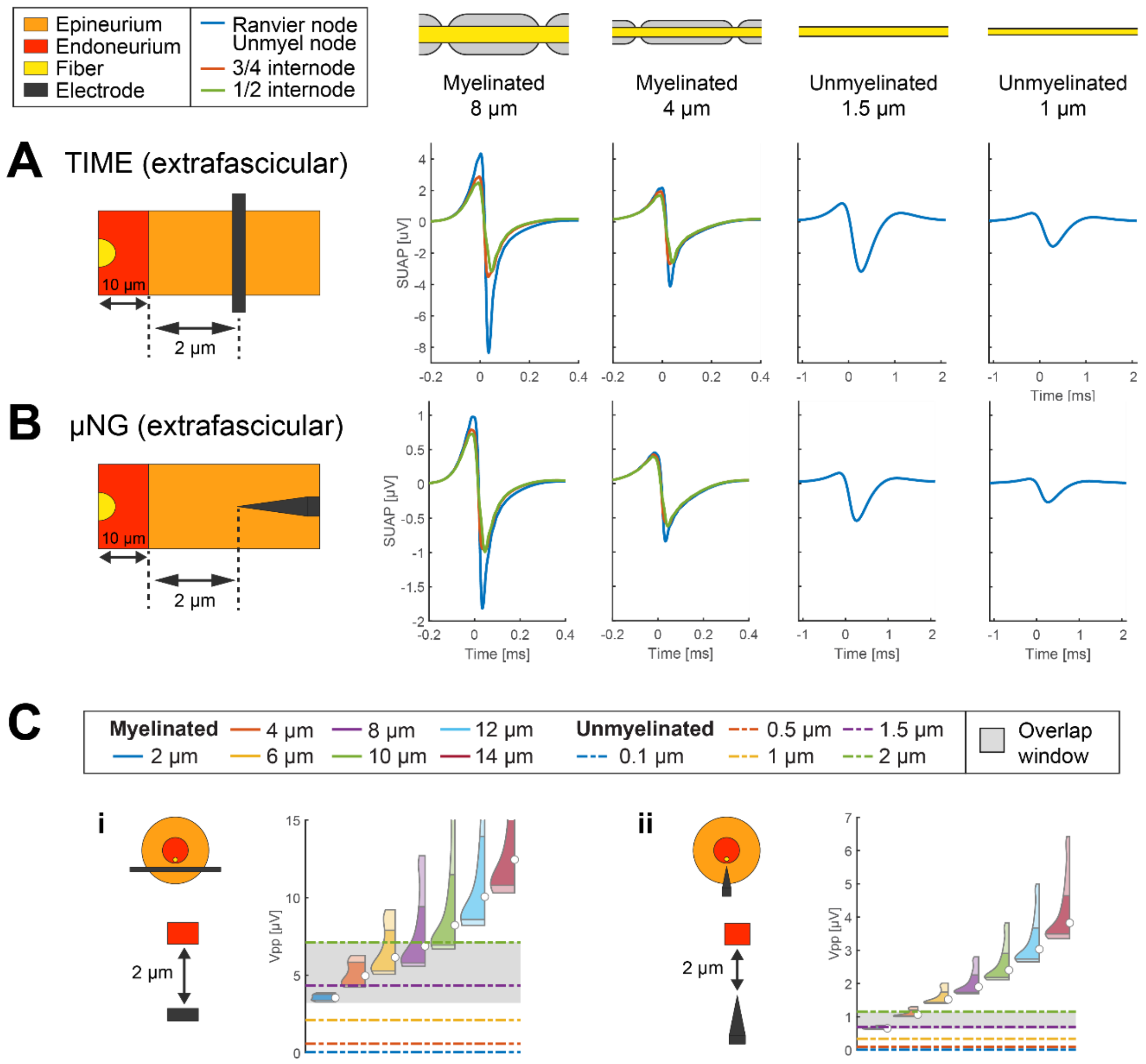

Figure S3. Relative amplitude of myelinated and unmyelinated SUAPs with electrodes close to fibers (extrafascicular insertions), related to Figure 3. The analyses in **Figure 3** are shown for the case of extrafascicular insertion of TIME and µNG.

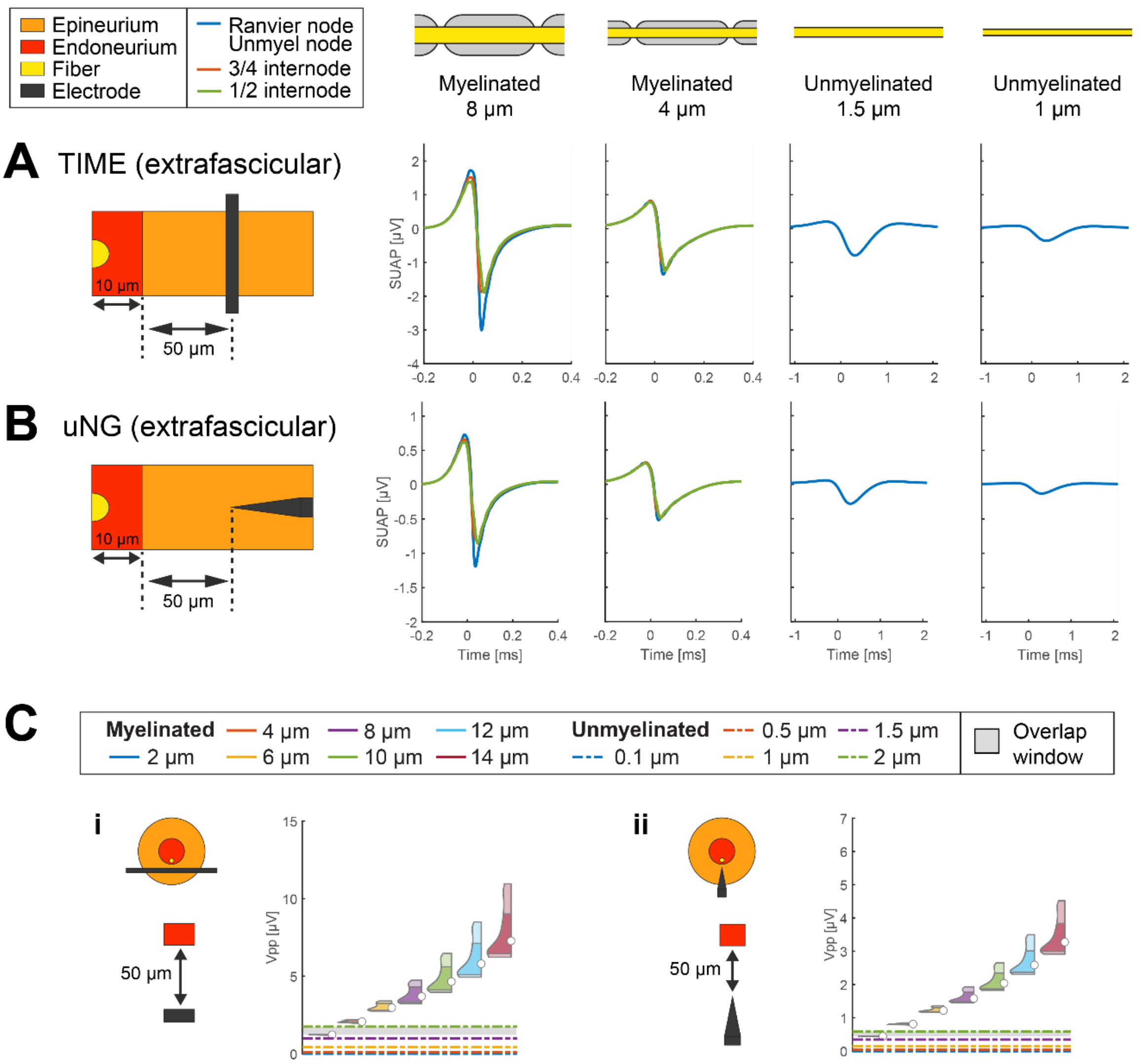

Figure S4. Relative amplitude of myelinated and unmyelinated SUAPs with electrodes far from fibers (extrafascicular insertions), related to Figure 4. The analyses in **Figure 4** are shown for the case of extrafascicular insertion of TIME and µNG.

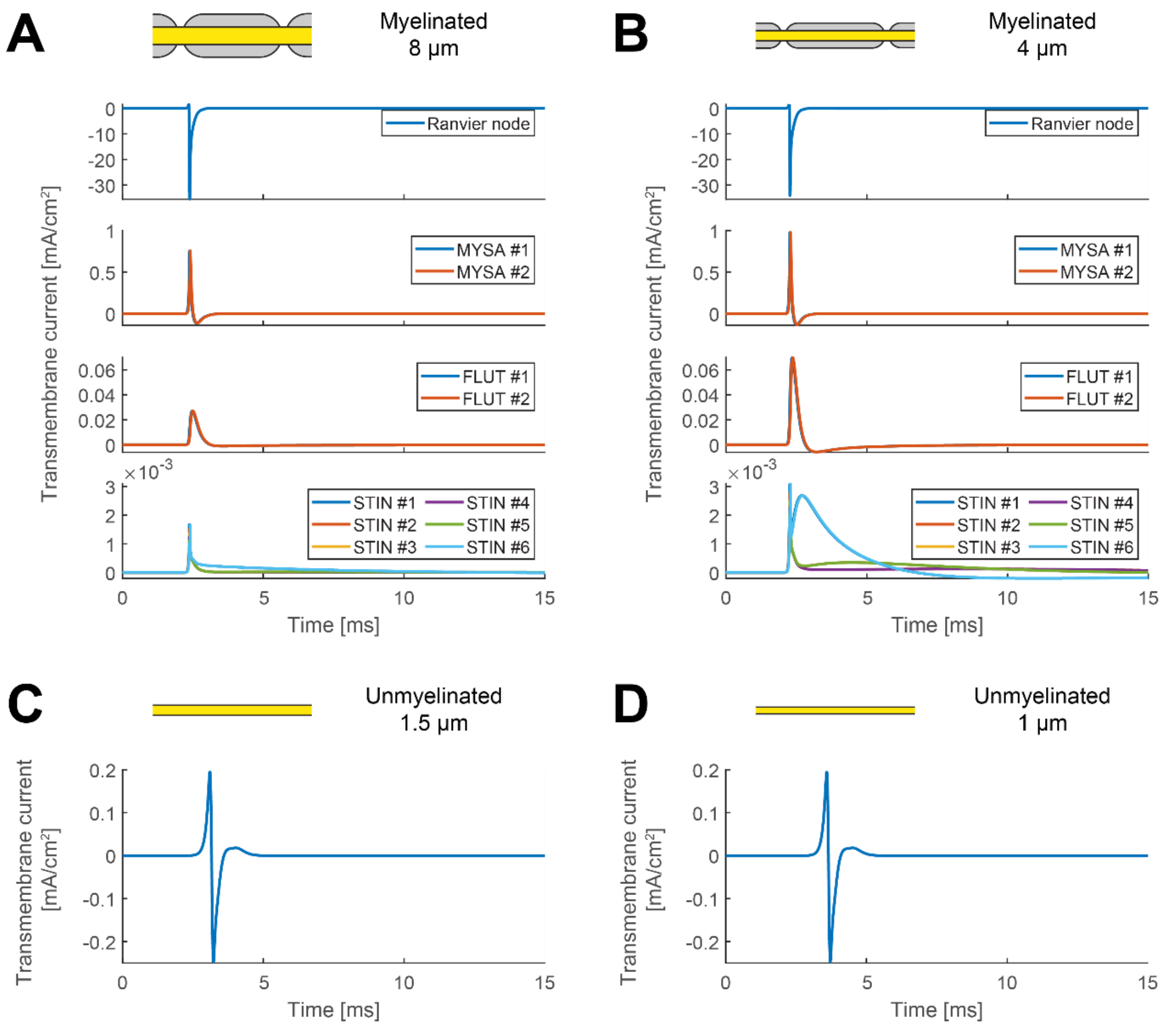

Figure S5. Transmembrane currents underlying spike propagation in myelinated and unmyelinated fibers, related to Discussion. A) 8 μm-myelinated fiber, B) 4 μm-myelinated fiber, C) 1.5 μm-unmyelinated fiber, and D) 1 μm-unmyelinated fiber. For myelinated fibers, the transmembrane currents are shown at each compartment of one internodal segment of the MRG model, in order: Ranvier node, MYSA #1, FLUT #1, STIN #1, STIN #2, STIN #3, STIN #4, STIN #5, #STIN #6, FLUT #2, MYSA #2. We note that such compartments differ in their lengths (see **Table S2**).

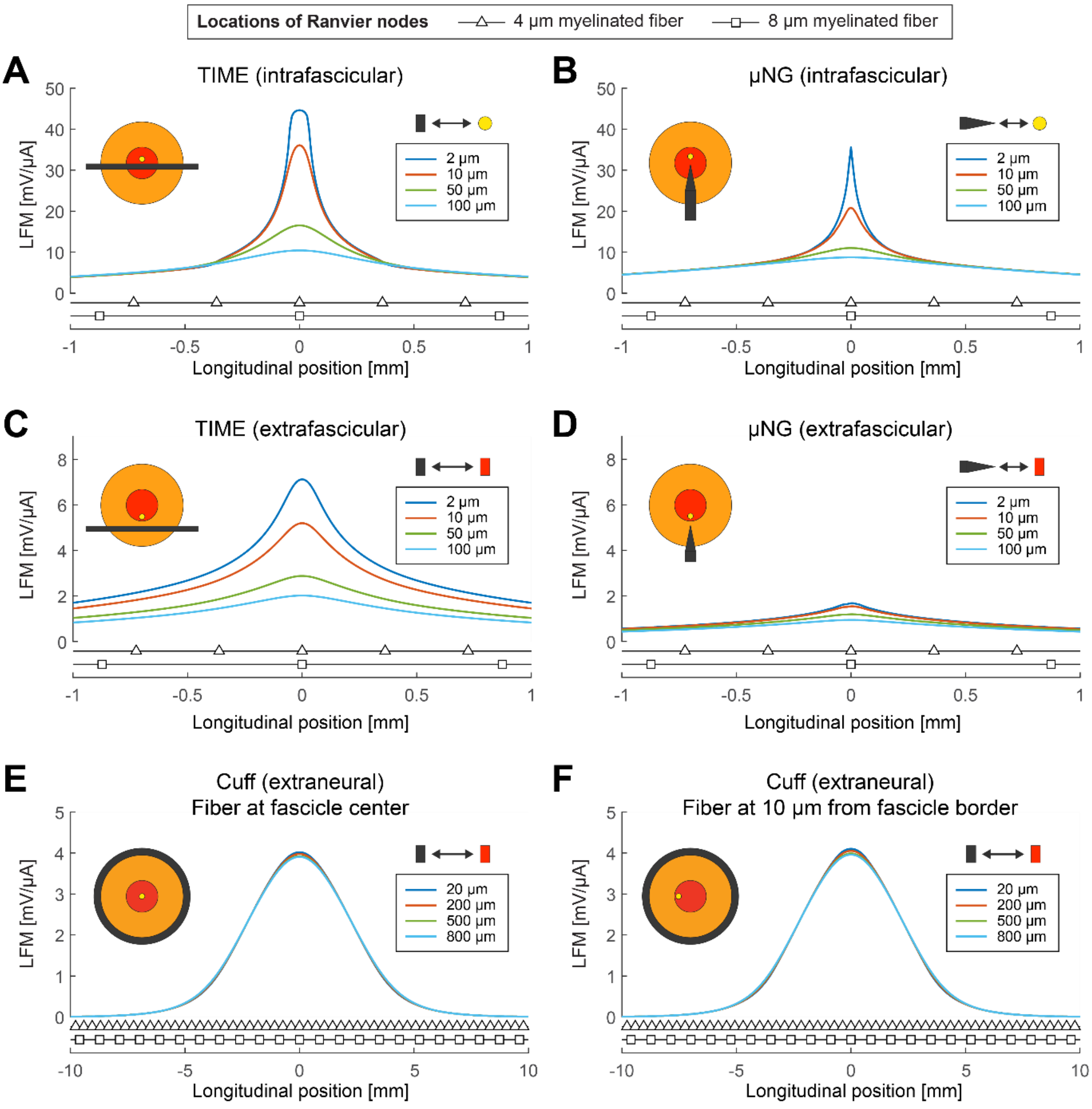

Figure S6. Lead-field matrix evaluated along a fiber with the electrodes placed at different distances, related to Discussion. A) intrafascicular TIME, B) intrafascicular μNG, C) extrafascicular TIME, D) extrafascicular μNG, E) cuff, and F) cuff with the fiber displaced to 10 μm from the fascicle boundary. Colors encode different fiber-to-electrode distances (for intrafascicular electrodes) or fascicle-to-electrode distances (for extrafascicular electrodes and cuff). Markers denote the position of Ranvier nodes in two myelinated fibers with 4 μm and 8 μm diameters.

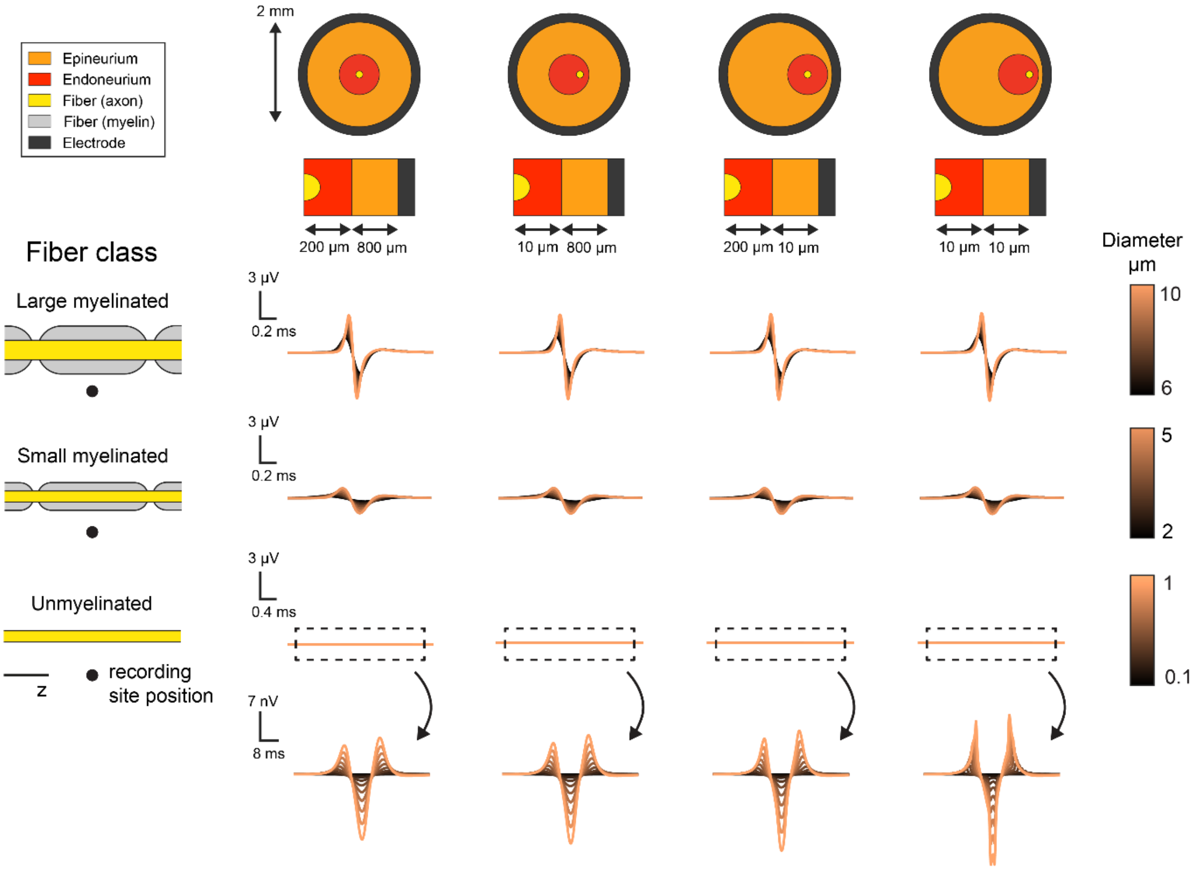

Figure S7. SUAPs recorded by the cuff with centered or eccentric placements of the fiber and the fascicle, related to Discussion. The analysis of SUAPs in **Figure 1G** is repeated for the cuff with: (first column) centered fiber and fascicle, (second column) eccentric fiber but centered fascicle, (third column) centered fiber but eccentric fascicle, and (fourth column) eccentric fiber and fascicle. We refer to the illustrations for the precise relative position of fiber, fascicle, and electrode.

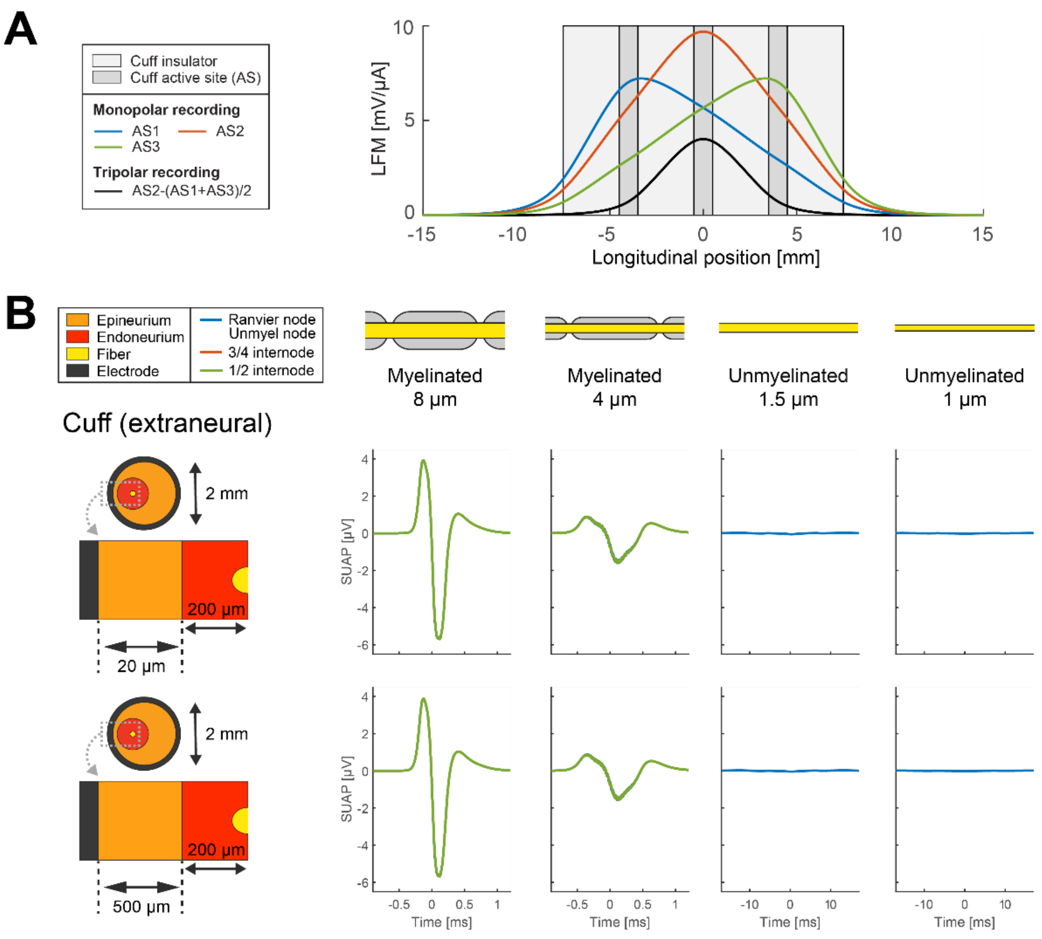

Figure S8. SUAPs recorded by the cuff in monopolar configuration, related to Discussion. A) Comparison of the lead-field matrix values along a fiber in the case of monopolar and tripolar recording configurations. B) Replication of the analyses in **Figure 3C** and **Figure 4C** using the central site of the cuff as recording site in monopolar configuration.

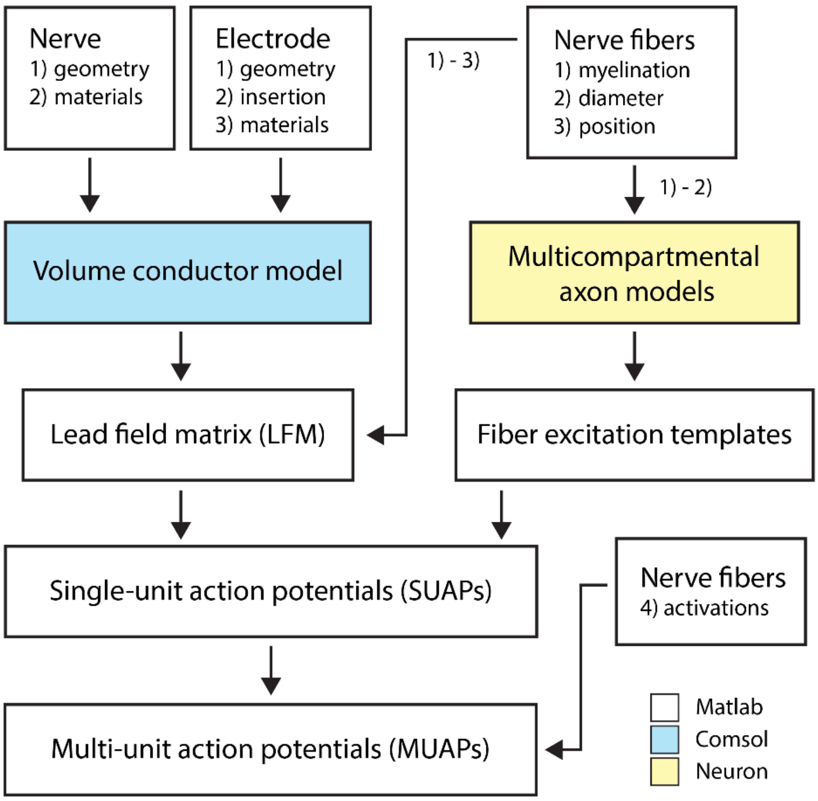

Figure S9. Flowchart of the modeling workflow used to simulate peripheral recordings, related to STAR Methods. We refer to STAR Methods for further details.

Humans					
Nerve	PNS division	# MY	# UM	% UM	Reference
Vagus (cervical)	Parasympathetic	18353	78023	81.0	[1] (n=30)
Vagus (abdominal)	Parasympathetic	1736	56139	97.0	[1] (n=10)
Greater splanchnic (preganglionic)	Sympathetic	6286	3951	38.6	[2] (n=1)
Greater splanchnic (postganglionic)	Sympathetic	4363	14867	77.3	[2] (n=1)
Tibial	Somatic	2001	4603	69.7	[3] (n=3)
Sural	Somatic	5858	22367	79.2	[4] (n=6)
Median (palmar cutaneous branch)	Somatic	563	2112	79.0	[5] (n=5)
Ulnar (dorsal cutaneous branch)	Somatic	-	-	90.6	[6] (n=14)

Rats					
Nerve	PNS division	# MY	# UM	% UM	Reference
Vagus (cervical)	Parasympathetic	8109	36409	81.8	[7] (n=10)
Vagus (abdominal)	Parasympathetic	72	9795	99.3	[8] (n=4)
		58	11077	99.5	[9] (n=30)
Pelvic splanchnic	Parasympathetic	979	4134	80.8	[10] (n=10)
Hypogastric	Sympathetic	218	1359	86.2	[10] (n=10)
Pudendal	Somatic	1741	3714	68.1	[10] (n=10)
Sciatic	Somatic	7588	18429	70.8	[11] (n=3)
		8022	16976	67.9	[12] (n=11)
Tibial	Somatic	4388	8433	69.4	[11] (n=3)
Sural	Somatic	1045	3879	78.8	[11] (n=3)
Peroneal	Somatic	1808	4100	65.8	[11] (n=3)

Table S1. Fiber composition of nerves relevant for neuroprosthetics and bioelectronic medicine applications, related to Introduction. The count of myelinated and unmyelinated fibers is reported, together with the percentage of unmyelinated fibers. Data refers to human (top) and rat (bottom) specimens. Data from specimens of left and right nerves were averaged. Abbreviations: PNS = peripheral nervous system, MY = myelinated fibers, UM = unmyelinated fibers.

Fiber	Internodal lengths						
Diameter [μm]	Total [μm]	Ranvier [%]	MYSA #1 [%]	FLUT #1 [%]	STIN #1-#6 [%]	FLUT #2 [%]	MYSA #2 [%]
2	200	0.50	1.50	5.88	84.74	5.88	1.50
4	362	0.28	0.83	6.21	85.65	6.21	0.83
6	558	0.18	0.54	5.71	87.33	5.71	0.54
8	873	0.11	0.34	4.58	90.04	4.58	0.34
10	1122	0.09	0.27	4.16	91.05	4.16	0.27
12	1306	0.08	0.23	4.00	91.47	4.00	0.23
14	1423	0.07	0.21	3.96	91.60	3.96	0.21

Table S2. Spatial discretization of myelinated fibers (MRG model), related to Discussion and STAR Methods. The table shows, for selected fiber diameters (first column), the lengths of the internode (second column) and its compartments (third-eighth columns). The lengths of the fiber compartments (Ranvier node, MYSA #1, FLUT #1, STIN #1-#6, FLUT #2, MYSA #2) are normalized with respect to the total length of the internode. Note that the six STIN compartments are collectively considered in the length count.

References

[1] H. H. Hoffman and H. N. Schnitzlein, “The numbers of nerve fibers in the vagus nerve of man,” *The Anatomical Record*, vol. 139, no. 3, 1961, doi: 10.1002/ar.1091390312.

[2] A. Kuntz, H. H. Hoffman, and E. M. Schaeffer, “Fiber components of the splanchnic nerves,” *The Anatomical Record*, vol. 128, no. 1, 1957, doi: 10.1002/ar.1091280111.

[3] A. E. Hines, H. Birn, P. S. Teglbjærg, and T. Sinkjær, “Fiber type composition of articular branches of the tibial nerve at the knee joint in man,” *Anatomical Record*, vol. 246, no. 4, 1996, doi: 10.1002/(SICI)1097-0185(199612)246:4<573::AID-AR18>3.0.CO;2-L.

[4] J. M. Jacobs and S. Love, “Qualitative and quantitative morphology of human sural nerve at different ages,” *Brain*, vol. 108, no. 4, 1985, doi: 10.1093/brain/108.4.897.

[5] U. Ahčan, Z. M. Arnež, F. F. Bajrović, A. Hvala, and P. Zorman, “Nerve fibre composition of the palmar cutaneous branch of the median nerve and clinical implications,” *British Journal of Plastic Surgery*, vol. 56, no. 8, 2003, doi: 10.1016/j.bjps.2003.08.012.

[6] A. L. C. R. D. Oliveira, V. P. S. Fazan, W. Marques, and A. A. Barreira, “Dorsal cutaneous branch of the ulnar nerve: A light and electron microscopy histometric study,” *Journal of the Peripheral Nervous System*, vol. 16, no. 2, 2011, doi: 10.1111/j.1529-8027.2011.00326.x.

[7] N. Soltanpour and R. M. Santer, “Preservation of the cervical vagus nerve in aged rats: Morphometric and enzyme histochemical evidence,” *Journal of the Autonomic Nervous System*, vol. 60, no. 1–2, 1996, doi: 10.1016/0165-1838(96)00038-0.

[8] G. Gabella and H. L. Pease, “Number of axons in the abdominal vagus of the rat,” *Brain Research*, vol. 58, no. 2, 1973, doi: 10.1016/0006-8993(73)90015-2.

[9] J. C. Prechtl and T. L. Powley, “The fiber composition of the abdominal vagus of the rat,” *Anatomy and Embryology*, vol. 181, no. 2, 1990, doi: 10.1007/BF00198950.

[10] C. E. Hulsebosch and R. E. Coggeshall, “An analysis of the axon populations in the nerves to the pelvic viscera in the rat,” *Journal of Comparative Neurology*, vol. 211, no. 1, 1982, doi: 10.1002/cne.902110102.

[11] H. Schmalbruch, “Fiber composition of the rat sciatic nerve,” *The Anatomical Record*, vol. 215, no. 1, pp. 71–81, 1986, doi: 10.1002/ar.1092150111.

[12] J. Castro, P. Negredo, and C. Avendaño, “Fiber composition of the rat sciatic nerve and its modification during regeneration through a sieve electrode,” *Brain Research*, vol. 1190, no. 1, 2008, doi: 10.1016/j.brainres.2007.11.028.
